# ﻿New species of *Sticta* (lichenised Ascomycota, lobarioid Peltigeraceae) from Bolivia suggest a high level of endemism in the Central Andes

**DOI:** 10.3897/mycokeys.92.89960

**Published:** 2022-09-13

**Authors:** Emilia Anna Ossowska, Bibiana Moncada, Martin Kukwa, Adam Flakus, Pamela Rodriguez-Flakus, Sandra Olszewska, Robert Lücking

**Affiliations:** 1 Department of Plant Taxonomy and Nature Conservation, Faculty of Biology, University of Gdańsk, Wita Stwosza 59, PL-80-308 Gdańsk, Poland University of Gdańsk Gdańsk Poland; 2 Licenciatura en Biología, Universidad Distrital Francisco José de Caldas, Cra. 4 No. 26D-54, Torre de Laboratorios, Herbario, Bogotá D.C., Colombia Universidad Distrital Francisco José de Caldas Bogotá Colombia; 3 Research Associate, Science & Education, The Field Museum, 1400 South Lake Shore, Chicago, IL 60605, USA Science & Education, The Field Museum Chicago United States of America; 4 Botanischer Garten, Freie Universität Berlin, Königin-Luise-Straße 6–8, 14195 Berlin, Germany Freie Universität Berlin Berlin Germany; 5 W. Szafer Institute of Botany, Polish Academy of Sciences, Lubicz 46, PL-31-512 Kraków, Poland W. Szafer Institute of Botany, Polish Academy of Sciences Krakow Poland; 6 10th High School in Gdynia, Władysława IV, PL-81-384 Gdynia, Poland 10th High School in Gdynia Gdynia Poland

**Keywords:** lichens, *Lobarioideae*, molecular barcoding, pigments

## Abstract

Six species of *Sticta* are described as new to science on the basis of material from Bolivia and supported by phylogenetic analysis of the fungal ITS barcoding marker. The species were resolved in all three of the clades (I, II, III) widespread and common in the Neotropics, as defined in an earlier study on the genus. Comparison with material from neighbouring countries (i.e. Colombia, Ecuador, Peru) suggests that these new species may be potentially endemic to the Bolivian Yungas ecoregion. For each species, a detailed morphological and anatomical description is given. *Stictaamboroensis* Ossowska, Kukwa, B. Moncada & Lücking is a medium-sized green-algal species with laminal to submarginal apothecia with hirsute margins and with light to dark brown lower tomentum. *Stictaaymara* Ossowska, Kukwa, B. Moncada, Flakus, Rodriguez-Flakus & Lücking is a comparatively small cyanobacterial taxon with *Nostoc* as photobiont, laminal, richly branched, aggregate isidia and a golden to chocolate-brown lower tomentum. The medium-sized, cyanobacterial *S.bicellulata* Ossowska, Kukwa, B. Moncada & Lücking has cyanobacterial photobiont, bicellular ascospores, apothecia with white to golden-brown hairs on the margins, K+ violet apothecial margin (ring around disc) and epihymenium and a white to dark brown lower tomentum. In contrast, the green-algal species, *S.carrascoensis* Ossowska, Kukwa, B. Moncada & Lücking is characterised by its large size, apothecia with dark brown hairs on the margins and a yellow medulla. The cyanobacterial *S.catharinae* Ossowska, B. Moncada, Kukwa, Flakus, Rodriguez-Flakus & Lücking forms stipitate thalli with *Nostoc* as photobiont, abundant, laminal to submarginal apothecia and a golden-brown lower tomentum. Finally, the cyanobacterial *S.pseudoimpressula* Ossowska, Kukwa, B. Moncada & Lücking produces laminal apothecia with an orange-yellow line of pruina along the margins which reacts K+ carmine-red. In addition to the six new Bolivian taxa, the cyanobacterial *S.narinioana* B. Moncada, Ossowska & Lücking is described as new from Colombia and it represents the closely-related sister species of the Bolivian *S.aymara*; it differs from the latter largely in the marginal instead of laminal isidia.

## ﻿Introduction

Bolivia is one of two landlocked countries in South America, besides Paraguay, being located almost in the centre of the continent. Situated in the Neotropics, it encompasses a diversity of ecosystems, with twelve ecoregions and twenty-three sub-regions (Ibisch and Mérida 2004; Moraes and Sarmiento 2019). This ecogeographical diversity is the reason why Bolivia harbours one of the highest biodiversity levels in the world (Feuerer et al. 1998; Josse et al. 2003). About 15000 vascular plant species have been documented, over 2000 of which are endemic (Jørgensen et al. 2014; Meneses et al. 2015). An exceptionally high diversity is also observed in lichens and lichenicolous fungi (e.g. Flakus and Lücking 2008; Kukwa and Flakus 2009; Flakus et al. 2011, 2012, 2013, 2016, 2019; Oset and Kukwa 2012; Guzow-Krzemińska et al. 2019). Yet, the number of species reported from Bolivia is about 70% lower than the estimated number (Lücking et al. 2009; Rodriguez de Flakus et al. 2016).

Amongst lichenised fungi, *Sticta* (Schreb.) Ach. is one of the genera for which the diversity in Bolivia is certainly underestimated. Published records are based almost exclusively on classical taxonomy and use outdated concepts of largely widely-distributed taxa and only two recent papers dealing with Bolivian species have employed a phylogenetic approach (Moncada et al. 2014a; Ossowska 2021). Integrative taxonomy, based on molecular data and morphological and anatomical information, have substantially refined taxon concepts in this genus, revealing taxa with similar morphology and anatomy, so-called ‘morphodemes’, to represent multiple species (e.g. Moncada 2012; Moncada et al. 2013a, b, 2020, 2021a, b, c; Widhelm et al. 2018; Mercado-Díaz et al. 2020). For example, several previously unrecognised species have recently been described in the *S.weigelii* (Ach.) Vain. morphodeme – i.e. cyanobacterial forms with marginal isidia, such as *S.andina* B. Moncada, Lücking & Sérus. and *S.scabrosa* B. Moncada, Merc.-Díaz & Bungartz, which are even more abundant than *S.weigelii* s.str. (Moncada et al. 2021a).

Modern approaches have also contributed to the discovery or revised definition of endemic taxa in different regions; for example, *S.aongstroemii* Dal Forno, B. Moncada & Lücking, endemic to south-eastern Brazil, *S.borinquensis* Merc.-Díaz & Lücking, endemic to Puerto Rico and *S.damicornis* (Sw.) Ach., a Caribbean endemic (Dal Forno et al. 2018; Moncada et al. 2018; Mercado-Díaz et al. 2020). Consequently, the number of formally described *Sticta* species is less than half the global estimate suggested by Moncada et al. (2013b, 2021a, b, c) and many new species may still remain unrecognised, especially in tropical ecosystems in South America and elsewhere, including Bolivia.

Here, we present the findings of a molecular revision of material of *Sticta* collected in Bolivia, integrating morphological and anatomical data, which resulted in the discovery of six new species, formally described in this study and an additional species from Colombia, closely related to one of the new Bolivian taxa. Their characteristics are elaborated in detail and a discussion on the potentially endemic occurrence of the new Bolivian species in the Yungas ecoregion of Bolivia is discussed.

## ﻿Materials and methods

### ﻿Taxon sampling

*Sticta* specimens were collected between 2010 and 2014 during fieldwork in the Yungas and Tucumano-Boliviano region. The collected material is deposited in KRAM, LPB and UGDA for the Bolivian specimens and in B and UDBC for the Colombian specimens. Morphology and anatomy were examined in Gdańsk and in Berlin, using Nikon SMZ800N and LEICA Zoom 2000 dissecting microscopes and ZEISS Axioskop compound microscopes, following the examination of characters as proposed by Moncada (2012) and Moncada et al. (2014a) and focusing on potentially diagnostic features at species level. Furthermore, we examined selected specimens from hitherto unpublished material collected in Colombia or other regions that turned out to be phylogenetically related to the Bolivian material. Spot reactions were performed with K (potassium hydroxide solution), C (sodium hypochlorite solution), Pd (para-phenylenediamine) and KC (K followed by C on the same thallus fragments) and secondary compounds were further analysed using thin-layer chromatography (TLC) in solvents A and C (Orange et al. 2001).

### ﻿DNA extraction, PCR amplification and sequencing

A total of 11 new specimens of *Sticta* from Bolivia and three from Colombia were used for the molecular study. Additionally, 194 ITS rDNA *Sticta* sequences were downloaded from GenBank, representing the monophyletic assembly of clades I, II and III as defined by Widhelm et al. (2018), using *S.macrothallina* as outgroup; the downloaded sequences were complemented by some previously unpublished sequences from Colombian material (Suppl. material 1: Table S1). For new DNA extractions from Bolivian material, two separate thallus fragments were taken from each specimen to allow cross-check of the results and account for potential sample contamination.

Total genomic DNA was extracted using the Sherlock AX Plant kit (A&A Biotechnology, Poland) and the Plant & Fungi DNA Purification Kit (Eurx, Poland), following the manufacturers’ protocol. Fungal ITS rDNA was amplified using the primers ITS1F and ITS4A (White et al. 1990; Gardes and Bruns 1993). The same primers were used for sequencing. PCR was carried out in a volume of 25 μl using 12.5 μl of Start-Warm HS-PCR Mix Polymerase (A&A Biotechnology, Poland), 1.0 μl of 10 μM of each primer, 1.0 μl of dimethyl sulphoxide (DMSO), 3.0 μl of template DNA (~ 10–100 ng) and water. The following PCR cycling parameters were applied: an initial denaturation at 94 °C for 3 min and 33 cycles of: 94 °C for 30 sec; annealing at 52 °C for 45 sec; extension at 72 °C for 1 min and final extension at 72 °C for 10 min. The PCR products were purified using PCR Clean Up System (Promega, US) and Clean-Up (A&A Biotechnology, Poland). The cleaned DNA was sequenced using Macrogen sequencing service (http://www.macrogen.com).

### ﻿Sequence alignment and phylogenetic analysis

The obtained sequences were aligned with available sequences of the genus *Sticta* (Suppl. material 1: Table S1; Suppl. material 2: File S1), using a previous master alignment (Moncada et al. 2020). The new sequences were joined to the existing alignment using MAFFT 7.164 with the “--add” option (Katoh and Frith 2012; Katoh and Standley 2013), with subsequent manual inspection in BioEdit 7.0.9 (Hall 2011). Phylogenetic analysis was performed using Maximum Likelihood in RAxML 8.2.0 (Stamatakis 2014) on the CIPRES Science Gateway (Miller et al. 2010), with non-parametric bootstrapping using 400 pseudoreplicates (based on an automated saturation criterion) under the GTRGAMMA model. Trees were visualised in FigTree 1.4.2 (Drummond and Rambaut 2007). After initial analysis of the full taxon set containing 1,049 terminals, the alignment was reduced to fully-identified taxa with valid names (except for one case where Bolivian samples were most closely related to a hitherto unnamed taxon from Colombia), with 3–5(–10) selected accessions per species, for a total of 211 terminals and the phylogenetic analysis was repeated using the above approach.

## ﻿Results and discussion

**Figure 1.**
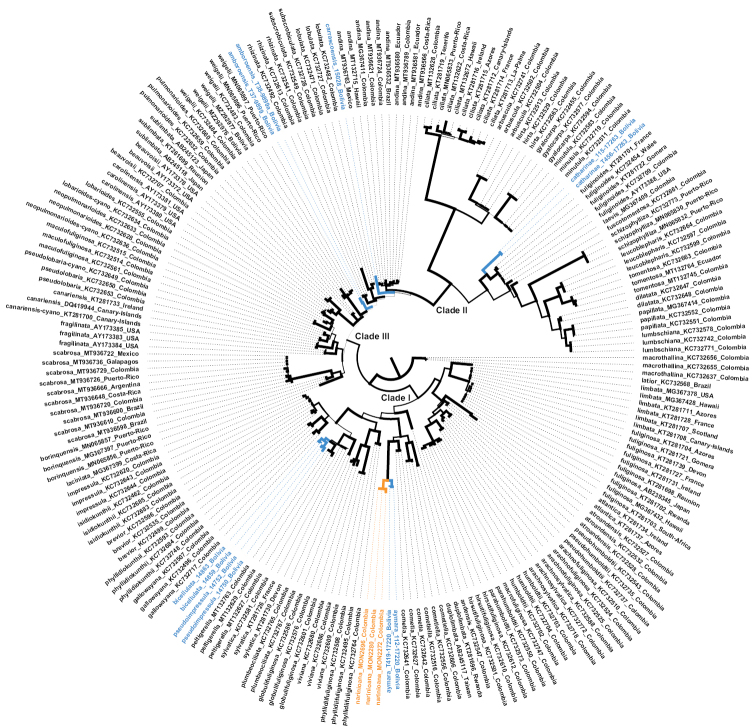
Best-scoring Maximum Likelihood tree of the *Sticta* clades I–III containing the new species from Bolivia (blue) and Colombia (orange), based on the fungal ITS barcoding marker. Supported clades are thickened. For a complete tree with individual support values, see Suppl. material 3: Fig. S1.

In the global phylogeny (Fig. 1; Suppl. material 3: Fig. S1), specimens from Bolivia formed five distinct lineages, suggesting six previously undescribed taxa, two of these representing closely-related sister taxa. The new species were recovered in all three clades (I, II, III) recognised by Widhelm et al. (2018) as common and widespread in the Neotropics: three in clade I (*fuliginosa* clade), one in clade II (*tomentosa* clade) and two in clade III (*weigelii* clade). With the use of the ITS barcoding marker only, the latter clade was not resolved as monophyletic, but formed a paraphyletic grade relative to clade II (Fig. 1; Suppl. material 3: Fig. S1). The first taxon, here named *Stictaaymara* and nested within the *S.cometia* clade, was recovered as sister to an undescribed species from Colombia, which is also described below as *S.narinioana*. Two other novel taxa, *S.bicellulata* and *S.pseudoimpressula*, fell within the *S.sylvatica* clade, closely related to *S.peltigerella* (Nyl.) Trevis. and *S.sylvatica* (Huds) Ach. In both of these taxa, *S.bicellulata* and *S.pseudoimpressula*, the apothecial margins (ring around disc) and epihymenium react with K, although the reactions differ in colour: it is violet in *S.bicellulata* and carmine-red in *S.pseudoimpressula*. This observation is noteworthy as it confirms the uniqueness of *Sticta* in Bolivia since, in most so far known taxa of the genus, the reaction with K is restricted to the medulla and membrane of the cyphellae. Further two new species, *S.amboroensis* and *S.carrascoensis*, were recovered close to *S.weigelii* and *S.andina*, respectively, but these placements were not supported in the backbone, so their exact phylogenetic relationships remain unresolved. Finally, *S.catharinae* was recovered as a novel taxon sister to the recently-described *S.fuliginoides* Magain & Sérus. (Fig. 1; Suppl. material 3: Fig. S1).

Analysis of morphological and anatomical features of these specimens and comparison with phylogenetically related and similar species supported their interpretation as new to science. These new species are characterised by various key features, described and discussed in detail along with overall morphological and anatomical characteristics in the ‘Taxonomy’ section below. At present, all species described below are known from only one site in Bolivia (although some are known from two collections); hence, it is difficult to estimate their frequency in this country. Nevertheless, it is reasonable to describe such species from single sites from such poorly studied countries, firstly due to the difficulty of exploring some areas of Bolivia and therefore the impossibility of splitting samples and secondly, because of the need for lichen conservation efforts in this country.

So far, the number of *Sticta* species known from Bolivia has not been critically assessed, most listings dating from the 19^th^ and early 20^th^ century (Nylander 1859, 1861; Rusby 1896; Herzog 1922, 1923). As a result, the recently assembled checklist (Rodriguez-Flakus et al. 2016) enumerates only 11 species, namely *S.damicornis* (Sw.) Ach., *S.dilatata* (Nyl.) Vain., *S.fuliginosa* (Dicks.) Ach., *S.isidiokunthii* B. Moncada & Lücking, *S.kunthii* Hook., *S.laciniata* (Sw.) Ach., *S.macrophylla* Bory ex Delise, *S.sinuosa* Pers., *S.tomentosa* (Sw.) Ach., *S.umbilicariiformis* Hochst. ex Flot. (Nylander 1861) and *S.weigelii*. Of these, the names *S.damicornis*, a Caribbean endemic, the paleotropical *S.macrophylla* and the African *S.umbilicariiformis* are certainly misapplications.

Recent research on *Sticta* (Moncada 2012; Moncada and Lücking 2012; Moncada et al. 2013a, b, 2014a, b, 2020, 2021a, b) has contributed to a much better understanding of the diversity of the genus and also facilitated further studies on this genus. As a consequence, *Sticta* has been more thoroughly assessed in other lichenologically poorly studied regions of the world (e.g. Simon et al. 2018; Widhelm et al. 2018; Mercado-Díaz et al. 2020; Moncada et al. 2020). In this respect, Bolivia remained as a ‘white spot’ on the map. The results presented here add to some previous works (Moncada et al. 2014a; Ossowska 2021) and substantially increase our knowledge of *Sticta* in Bolivia, cataloguing the true diversity of this genus in the country using more accurate delimitation of species supported by phylogenetic analyses. Yet unpublished data from our group, including a large number of still unnamed clades that require thorough revision including phenotype characters, suggest that the number of taxa present in Bolivia may be comparable to the diversity now known from Colombia (more than 200 species) (Moncada 2012; Moncada et al. 2013a, b, 2014a, b, 2020, 2021a).

Considering that the globally available sequence data of *Sticta* originate from nearly 30 different countries, it is notable that sequenced specimens from Bolivia mostly represent novel lineages, which suggests that these taxa may be endemic to the Yungas ecoregion in Bolivia. However, further studies in nearby regions are required to test this hypothesis. Endemic species of *Sticta* have been reported by other authors from various regions (e.g. Tønsberg and Goward 2001; Lendemer and Goffinet 2015; Dal Forno et al. 2018; Moncada et al. 2018, 2020; Widhelm et al. 2018). In Madagascar and the Mascarenes, for instance, where the genus has been very well sampled, 89% of the known species are endemic (Simon et al. 2018). A similarly high proportion has been reported from Puerto Rico, although Mercado-Díaz et al. (2020) noted that this number may be overestimated due to a lack of comparative data from neighbouring islands. The degree of endemism of *Sticta* in Hawaii is estimated at 69% (Moncada et al. 2020). While the large number of endemic taxa on the islands is related to their geographical isolation (Nash 2008), a high proportion of endemic lichens is also observed, for example, in isolated tropical mountains or in fog-induced lichen zones in coastal deserts, like Namibia (Aptroot 2008; Aptroot and Schumm 2011; Sparrius et al. 2017).

The putative endemism of the species described here is also supported by the fact that the specimens were collected from different sites located within the Yungas ecoregion on the north-eastern slopes of the Andes. This is an area situated at 1000–4200 m altitude and extends over 55,556 km. It is mainly covered by evergreen humid forest and includes several protected areas, such as Madidi and Cotapata National Parks (Ibisch and Mérida 2004; Moraes and Sarmiento 2019). Yungas is a species-rich area and has been considered a centre of endemism in Bolivia for different groups of organisms, such as algae, bryophytes and orchids (e.g. Kessler 1999; Ibisch and Mérida 2004; Moraes and Sarmiento 2019; Kolanowska et al. 2021; Kosecka et al. 2021).

A large number of putatively endemic taxa within *Sticta*, not only in Bolivia, but also in other regions, may account for the discrepancy between the described and expected number of species in this genus, with 200 species known vs. 500 predicted (Moncada et al. 2013a, b, 2021b; Mercado-Díaz et al. 2020). Our results from Bolivia increase the global number of *Sticta* by another six new species, thus slowly chipping away at this knowledge gap.

### ﻿Taxonomy

#### New species from Bolivia

##### ﻿ Sticta amboroensis

Taxon classificationFungiPeltigeralesPeltigeraceae

Ossowska, Kukwa, B. Moncada & Lücking sp. nov.

0C81B12C-338C-5FD9-8DDD-BFB015FD3B7B

 MB845385

Fig. 2

###### Diagnosis.

Differing from *S.subscrobiculata* in the larger thalli with abundant marginal cilia and marginal and laminal apothecia with veined lower surface and the thickness of the upper cortex with 60–80 μm.

**Figure 2.**
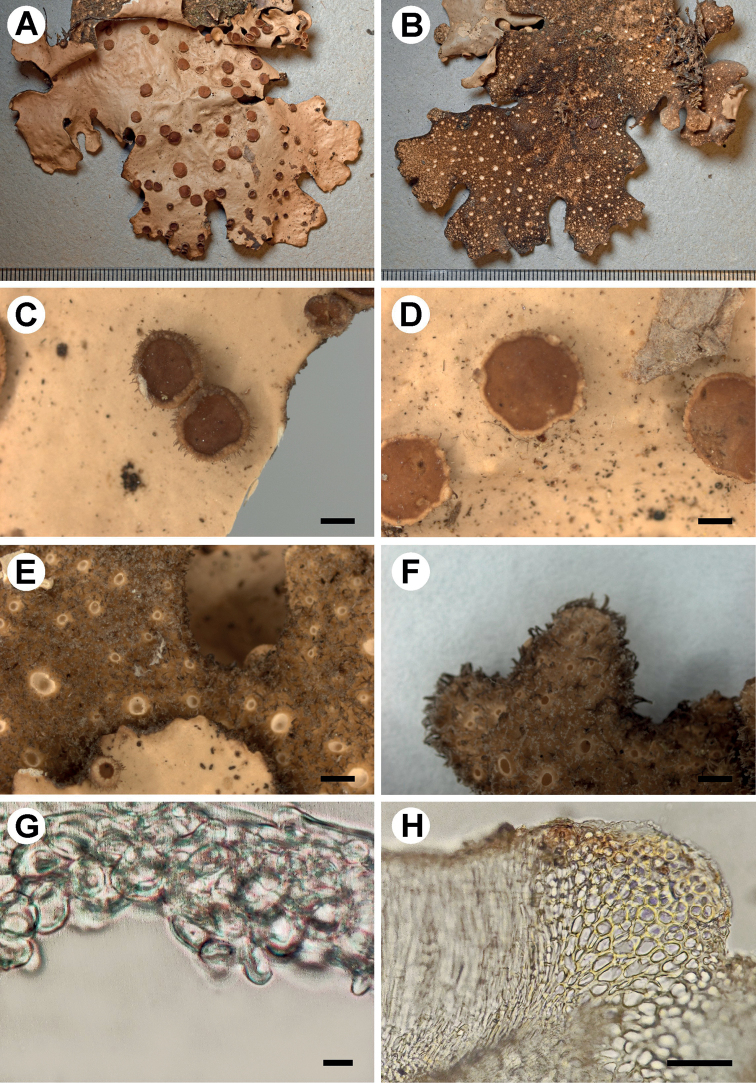
Morphology of *Stictaamboroensis* (holotype) **A** upper surface **B** lower surface **C–D** apothecia with hirsute margins **E** lower tomentum with cyphellae **F** marginal cilia **G** cyphella membrane **H** excipulum in section. Scale bars: 1 mm (**A–F**); 5 μm (**G**); 50 μm (**H**).

###### Type.

Bolivia. Dept. Santa Cruz; Prov. Florida, Parque Nacional Amboró, above la Yunga Village, senda los Helechos, near view point, 18°02'50"S, 63°54'50"W, elev. 2330 m, Yungas cloud forest with abundant tree ferns, corticolous, 08 June 2011, M. Kukwa 9899 (holotype UGDA, isotype LPB).

###### Description.

Primary photobiont a green alga. Stipe absent. Thallus irregular, up to 25 cm diam., moderately branched, with 3–5 branches per 5 cm radius, branching polytomous; lobes ligulate to laciniate, imbricate to adjacent, involute, with their apices rounded to obtuse and involute and their margins entire to sinuous, not thickened; lobe internodes (3–)7–10(–20) mm long, (4–)8–7(–18) mm broad; thallus coriaceous. Upper surface plane to rugose-pitted towards the centre, beige-brown with darker apices in the herbarium, shiny, with the brown marginal line; surface glabrous, without papillae, without pruina, but with irregular to indistinct, cream maculae, present in older parts of lobes; marginal cilia abundant, simple to fasciculated, light to dark brown, rarely white, up to 0.5 mm long. Apothecia abundant, principally submarginal to laminal, sparse to aggregated, subpedicellate, without pronounced invagination on lower side, up to 3 mm diam.; disc light brown to brown (mature) and yellow (young), shiny to matt; margin entire to crenate, hirsute, with brown hairs, abundant in young apothecia, sparse in old ones. Vegetative propagules absent. Lower surface with somewhat elevated, diffuse ridges, cream to brown towards the centre; primary tomentum dense, sparse towards the margin, thick but thinner towards the margin, spongy to fasciculate, soft, light brown to dark brown; secondary tomentum absent. Rhizines scarce, brown to white, up to 8 mm long. Cyphellae 1–20 per cm^2^ towards the thallus centre and 21–40 per cm^2^ towards the margin, scattered, rounded to irregular, urceolate with wide pore, erumpent to prominent, remaining below the level of the primary tomentum, with the margin raised and involute, cream-coloured, with tomentum; pore 0.5–1.5 mm diam.; basal membrane pruinose in appearance, white to cream, K+ pale yellow, C–, KC–, Pd–. Medulla lax to compact, white, K–, C–, KC–, Pd–. No substances detected by TLC.

Upper cortex paraplectenchymatous, 60–80 μm thick, consisting of 6–7 cell layers with cells 6–16 μm diam. (with smaller cells in outside parts of the cortex), their walls 3–5 μm thick and their lumina rounded to isodiametric, 4–15 μm diam. Photobiont layer 35–50 μm thick, its cells 5–8 μm diam. Medulla 150–220 μm thick, its hyphae 4–5 μm broad, without crystals. Lower cortex paraplectenchymatous, 30–45 μm thick, with 3–4 cell layers; cells 6–17 μm diam., their walls 2–7 μm thick. Hairs of lower primary tomentum up to 1 mm long, in fascicles of 12–20, hyphae unbranched, 5–6 μm wide with rugose walls, forming a brush-like head with free apices. Cyphella cavity up to 300 μm deep; compacted cells of basal membrane rarely with one papillae. Apothecia biatorine, up to 700 μm high, without distinct stipe; excipulum 125–175 μm broad, laterally with projecting hairs, 50 μm long, simple or in groups. Hymenium 100–110 μm high, K+ yellow; epihymenium up to 20 μm high, yellow-brown, K+ yellow intensifying, with gelatinous upper layer, ca. 5 μm high. Asci 6–8-spored, ascospores fusiform, 1–3-septate, 27–42 × 6–10 μm.

###### Habitat and distribution.

*Stictaamboroensis* is known only from one locality in Parque Nacional Amboró in Department Santa Cruz, where it grows on tree bark at altitude 2330 m.

###### Etymology.

The name refers the Parque Nacional Amboró, where the species was found.

###### Additional material examined.

Bolivia. Dept. Santa Cruz; Prov. Florida, Parque Nacional Amboró, above la Yunga Village, senda los Helechos, near view point, 18°02'50"S, 63°54'50"W, elev. 2330 m, Yungas cloud forest with abundant tree ferns, 08 June 2011, M. Kukwa 9899a (LPB, UGDA).

###### Notes.

*Stictaamboroensis* forms an isolated lineage not far from other green algal species, such as *S.pulmonarioides* B. Moncada & Coca and *S.subscrobiculata* (Nyl.) Gyeln. These species are characterised by a similar morphology, but clearly distinguished phylogenetically. In *S.subscrobiculata*, the lobes are sparsely branched and pleurotomous and the marginal cilia are sparse to absent, although a cilia-like extension of the lower tomentum is usually visible (Moncada 2012). In the case of *S.pulmonarioides*, thallus is smaller, up to 15 cm in diam., with sparse, mainly submarginal apothecia. In addition, the lower tomentum is sparse over the entire lower surface (Moncada et al. 2013a).

##### ﻿ Sticta aymara

Taxon classificationFungiPeltigeralesPeltigeraceae

Ossowska, Kukwa, B. Moncada, Flakus, Rodriguez-Flakus & Lücking sp. nov.

10515322-8238-5FCB-A5B9-B27353273FF1

 MB845386

Fig. 3

###### Diagnosis.

Differing from *S.narinioana* in the presence of laminal isidia and in the absence of apothecia, as well as the less densely arranged cyphellae.

**Figure 3.**
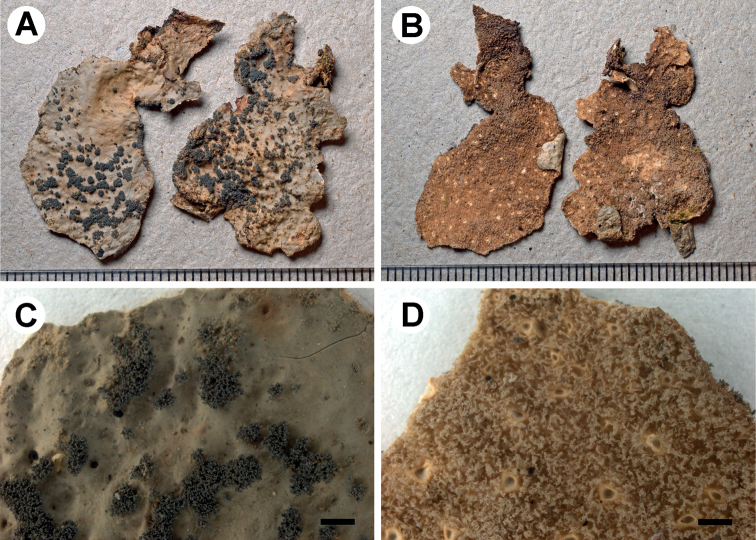
Morphology of *Stictaaymara* (holotype) **A** upper surface with isidia **B** lower surface showing shallow ridges (visible on right-hand lobe after removal of tomentum) **C** laminal and branched isidia **D** tomentum and cyphellae. Scale bars: 1 mm.

###### Type.

Bolivia. Dept. La Paz; Prov. Nor Yungas, Parque Nacional y Área Natural de Manejo Integrado Cotapata, near Urpuma colony, 16°13'20"S, 67°52'34"W, elev. 1989 m, Yungas montane forest, 30 June 2010, A. Flakus 17220 & P. Rodriguez-Flakus (holotype KRAM, isotype LPB).

###### Description.

Primary photobiont cyanobacterial (*Nostoc*). Stipe absent. Thallus orbicular to irregular, up to 5 cm diam., sparsely branched, with 0–2 branches per 5 cm radius, branching pleurotomous; lobes suborbicular to flabellate, interspaced to adjacent, plane to undulate, with their apices rounded and revolute and their margins entire to sinuous, not thickened; lobe internodes (1–)2–4(–7) mm long, (3–)5–6(–10) mm broad; thallus subcoriaceous. Upper surface smooth to pitted or rugose towards the centre, brownish-yellow with darker apices in the herbarium, shiny; surface glabrous, without papillae and pruina, but with irregular, scattered, yellow maculae; marginal cilia absent. Apothecia absent. Vegetative propagules present, abundant, in the form of isidia, predominantly laminal, aggregate, richly branched from the beginning, isidial branches cylindrical to coralloid, vertical, up to 0.6 mm long and 0.05 mm broad, darker than the thallus, grey, shiny; in cross-section, round or rarely slightly flattened. Lower surface with somewhat elevated, diffuse ridges, yellow to brown towards the centre; primary tomentum dense to the margin, thick but thinner towards the margin, spongy to fasciculate, soft, golden to chocolate; secondary tomentum present, arachnoid. Rhizines absent. Cyphellae sparse, 1–10 per cm^2^ towards the thallus centre and 1–20 per cm^2^ towards the margin, scattered, angular to irregular, urceolate with wide pore, prominent, remaining below the level of the primary tomentum, with the margin raised and involute, cream-coloured, with or without tomentum; pore 0.25–0.75 mm diam.; basal membrane ± smooth, white, K–, C–, KC–, Pd–. Medulla compact, cream, K–, C–, KC–, Pd–. No substances detected by TLC.

Upper cortex paraplectenchymatous, 15–40 μm thick, consisting of 2–3 cell layers with cells 7–18 μm diam. (with smaller cells in outside parts of the cortex), their walls 0.6–2 μm thick and their lumina rounded to isodiametric, 6–17 μm diam. Photobiont layer 25–70 μm thick, its cells 4–20 μm diam. Medulla 30–70 μm thick, its hyphae 2.5–6 μm broad, without crystals. Lower cortex paraplectenchymatous, 30–50 μm thick, with 3 cell layers; cells 6–20 μm diam., their walls 2–4 μm thick. Hairs of lower primary tomentum 150–400 μm long, in fascicles of more than 20, hyphae simple, septate with interlocked apices. Cyphella cavity up to 130 μm deep; cells of basal membrane without papillae or with single papillae. Apothecia not observed.

###### Habitat and distribution.

*Stictaaymara* is known only from the type locality in the Department La Paz, at an altitude of 1989 m.

###### Etymology.

The name refers the Aymara people in the Andes and Altiplano regions of South America who coined the term Yungas.

###### Notes.

Although this new taxon is known from a single collection only, we decided to describe it formally, as the material is well-developed and phylogenetically distinctive, shown by two sequences generated from different pieces of the specimen. *Stictaaymara* forms a sister clade with the also newly-described *S.narinioana* from Colombia (see below). Both taxa produce isidia, but in *S.narinioana*, they are concentrated along the thallus margins and horizontally orientated, while in *S.aymara*, they are laminal and upright. Moreover, sparse, submarginal apothecia, absent in *S.aymara*, were observed in *S.narinioana*. Cilia are absent in both taxa, but in *S.narinioana*, the white tomentum projects beyond the edge of the lobes and resembles cilia. The two species also differ in the abundance of cyphellae, which are more densely arranged in *S.narinioana*.

The presence of isidia is also characteristic for *S.isidiokunthii* B. Moncada & Lücking and *S.weigelii*, amongst other similar species (Moncada 2012). However, isidia in these species are mainly marginal and differ in colour. In *S.aymara* the isidia are grey, in *S.isidiokunthii*, greenish-brown to brown and in *S.weigelii*, blackish-brown. Moreover, the latter taxa are characterised by thalli larger than *S.aymara*, up to 10–15 cm in diam. Differences were also observed in the structure and colour of the lower surface. In *S.isidiokunthii*, it is uneven, beige to dark brown, while in *S.weigelii*, it is smooth to undulate, beige to red-brown (Moncada 2012; Moncada and Lücking 2012; Ossowska 2021). Additionally, *S.isidiokunthii* also produces laminal apothecia (Moncada and Lücking 2012). The medulla of both *S.isidiokunthii* and *S.weigelii* reacts with K, while in *S.aymara*, it is K negative.

The small size of the thalli, the presence of isidia and the absence of apothecia is also characteristic of *S.viviana* A. Suárez & Lücking. However, this taxon has a dark brown, scrobiculate to faveolate upper surface with cream-coloured maculae. Furthermore, the lower part is rugose to undulating, rather than ridged-veined as in *S.aymara*. The medulla of *S.viviana* is K+ orange-yellow and the cyphellae are K+ yellow. The latter species is known from Colombia and Costa Rica (Moncada 2012; Suárez and Lücking 2013; Moncada et al., unpub.). *Stictaaymara* and *S.viviana* are phylogenetically only distantly related (Fig. 1; Suppl. material 3: Fig. S1).

##### ﻿ Sticta bicellulata

Taxon classificationFungiPeltigeralesPeltigeraceae

Ossowska, Kukwa, B. Moncada & Lücking sp. nov.

8FEE5013-C6CE-592C-8E1F-6570F7C6ECB0

 MB845387

Fig. 4

###### Diagnosis.

Differing from *S.pseudoimpressula* in the predominantly bicellular spores and the absence of secondary tomentum and the K+ violet (instead of carmine-red) reaction of the apothecial atraquinone.

**Figure 4.**
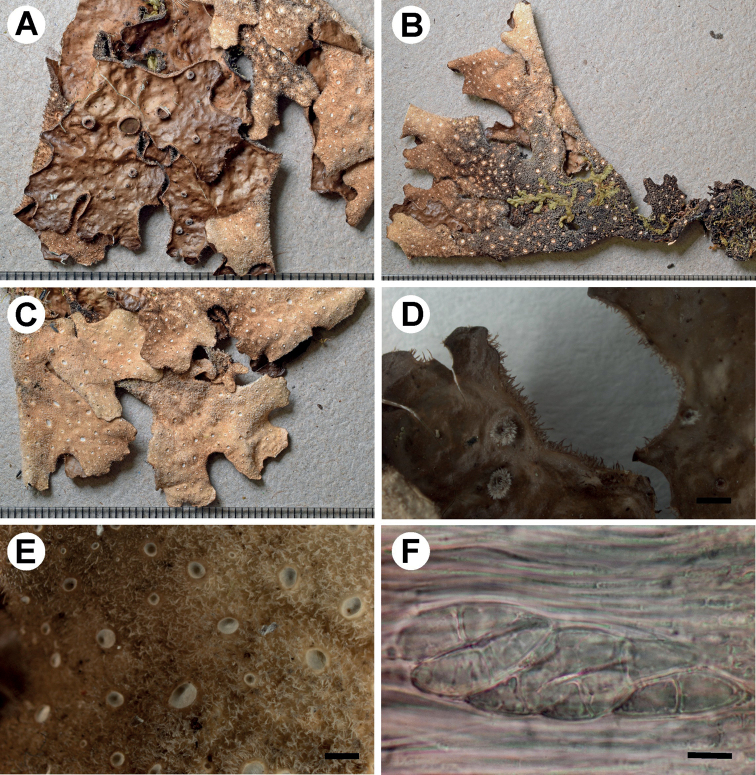
Morphology of *Stictabicellulata* (holotype) **A** enlarged part of thallus showing pitted to rugose upper surface and laminal apothecia **B** lower surface **C** enlarged part of lower surface of the thallus **D** apothecia with hirsute margins and cilia at the lobe margins **E** tomentum and cyphellae **F** ascus with 1-septate ascospores. Scale bars: 1 mm (**A–E**); 10 μm (**F**).

###### Type.

Bolivia. Dept. La Paz; Prov. Franz Tamayo, Parque Nacional y Área Natural de Manejo Integrado Madidi, near Keara Bajo, 14°41'59"S, 69°04'34"W, elev. 3290 m, open area with shrubs and scattered trees, Ceja de Monte Inferior (Altimontano), on shrubs, 17 Nov 2014, M. Kukwa 14859 (holotype UGDA, isotype LPB).

###### Description.

Primary photobiont cyanobacterial (*Nostoc*). Stipe absent. Thallus irregular to suborbicular, up to 10 cm diam., moderately branched, with 3–5 branches per 5 cm radius, branching polytomous; lobes laciniate to flabellate, adjacent, involute to weakly canaliculate, with obtuse to truncate, plane to revolute apices and their margins entire, slightly thickened; lobe internodes (3–)6–8(–20) mm long, (4–)7–10(–13) mm broad; thallus coriaceous. Upper surface pitted to shallowly scrobiculate to rugose, light brown with darker apices in the herbarium, shiny, with the marginal line in the same colour; surface glabrous, without papillae and pruina, without maculae; marginal cilia present, about 0.5 mm, abundant to scarce, white to golden brown, agglutinated. Apothecia scarce, laminal, dispersed, subpedicellate, with pronounced invagination on lower side, up to 2.5 mm diam.; disc orange-brown (in young apothecia) to brown or greenish (in older apothecia) due to the presence of pruina, along the margin with an orange pigment; margin hirsute, with white to golden brown hairs. Vegetative propagules absent. Lower surface uneven, beige to light brown; primary tomentum dense to the margin, thick, but thinner towards the margin, spongy, soft, white to dark brown often with brown tips; secondary tomentum absent. Rhizines absent. Cyphellae 1–10 per cm^2^ towards the thallus centre and 21–40 per cm^2^ towards the margin, abundant, scattered, rounded to irregular, urceolate with wide pore, prominent, remaining below the level of the primary tomentum, with the margin raised and involute, white to brown coloured, without or with tomentum at the base; pore (0.25–)0.5–1(–1.5) mm diam.; basal membrane pruinose in the appearance, white, K– to K+ yellow, C–, KC–, Pd–. Medulla compact, white with yellow spots, K+ pale yellow, C–, KC–, Pd–. Apothecial margin (ring around disc) and epihymenium K+ violet. No substances detected by TLC in the thallus, unidentified anthraquinone in the apothecia.

Upper cortex paraplectenchymatous, 35–50 μm thick, consisting of 3–4 layers of cells 5–16 μm diam. (with smaller cells in outside parts of the cortex), their walls 1.5–3.5 μm thick and their lumina rounded to isodiametric, 4–15 μm diam. Photobiont layer 40–80 μm thick, its cells 5–10 μm diam. Medulla 35–50 μm thick, its hyphae 5 μm broad, without crystals. Lower cortex paraplectenchymatous, 25–35 μm thick, with 2–3 cell layers; cells 7–13 μm diam., their walls 2.5–5 μm thick. Hairs of lower primary tomentum 100–250 μm long, in fascicles of more than 20 when mature, simple to rarely branched hyphae, 5–6 μm broad, septate with free apices. Cyphella cavity up to 100 μm deep; cells of basal membrane loosely packed consisting of cells, without papillae or very rarely, with one papillae. Apothecia biatorine, up to 100–250 μm high, without a peduncle; excipulum 80–100 μm broad, laterally with projecting hairs, hairs simple, up to 110 μm long or in groups up to 300 μm long, hairs 4–6 μm broad, thick-walled, septate. Hymenium 100–112 μm high; epihymenium 12.5–20 μm high, orange-brown, with orange granules crystals, with thin gelatinous upper layer. Asci 6–8-spored, ascospores broadly fusiform, 1(–3)-septate, 30–41 × 9–12 μm.

###### Habitat and distribution.

The species is known from the Parque Nacional y Área Natural de Manejo Integrado Madidi, a protected area in the Department La Paz. It was found epiphytic at an elevation of 3290 m.

###### Etymology.

The epithet refers to the predominance of bicellular spores.

###### Additional material examined.

Bolivia. Dept. La Paz; Prov. Franz Tamayo, Parque Nacional y Área Natural de Manejo Integrado Madidi, near Keara Bajo, 14°41'59"S, 69°04'34"W, elev. 3290 m, open area with shrubs and scattered trees, Ceja de Monte Inferior (Altimontano), on shrubs, 17 Nov 2014, M. Kukwa 14863 (LPB, UGDA).

###### Notes.

*Stictabicellulata* is similar to *S.pseudoimpressula* (another species described below), but the main discriminating character in *S.bicellulata* is the septation of the ascospores, which are predominantly bicellular (only very few are 3-septate whereas in *S.pseudoimpressula*, only young ascospores are bicellular. Both taxa have irregular to suborbicular thalli, with laciniate to flabellate lobes, but the lobe apices in *S.bicellulata* are obtuse to truncate vs. orbicular in *S.pseudoimpressula*. Furthermore, *S.bicellulata* has a paler upper surface than *S.pseudoimpressula*. In both species, marginal cilia are present, but in *S.bicellulata*, they are agglutinated, white to golden brown vs. fasciculated, light brown to golden brown in *S.pseudoimpressula*. Apothecia in *S.bicellulata* are sparse in contrast to the abundant apothecia in *S.pseudoimpressula*. They also differ in the colour of the disc, which in both species is covered by a pruina. The margin and epihymenium react with K in both taxa, but in *S.bicellulata* the reaction is K+ violet and in *S.pseudoimpressula* K+ carmine-red, suggesting the presence of different anthraquinones. The lower surface in both species is uneven, but in *S.bicellulata*, the tomentum is thick, becoming thinner towards the margin and a secondary tomentum is absent. Conversely, in *S.pseudoimpressula*, the primary tomentum is consistently thick and long, with a secondary tomentum present and with rhizines. The cyphellae in these two taxa are similar in shape, but in *S.bicellulata*, their margins are raised and involuted.

Remarkably, the taxa form a sister group relationship, denoting the presence of apothecial anthraquinones as a synapormorphy, although apparently, the two species diverged to the point that the anthraquinones are of a different nature, as indicated by their different K+ reaction. This character appears to be rare in *Sticta*, but may also have been overlooked, as it is only obvious in a close-up of the apothecium.

The clade formed by *Stictabicellulata* and *S.pseudoimpressula* is closely related to *S.sylvatica* and *S.peltigerella* (Fig. 1; Suppl. material 3: Fig. S1). The latter two produce numerous isidia distributed over the entire surface of the thalli (Moncada 2012). *Stictasylvatica* is widespread, occurring in Europe, North and South America (Hodkinson et al. 2014), whereas *S.peltigerella* appears to be a Colombian endemic (Moncada 2012).

##### ﻿ Sticta carrascoensis

Taxon classificationFungiPeltigeralesPeltigeraceae

Ossowska, Kukwa, B. Moncada & Lücking sp. nov.

ADDA34D5-9847-5E48-B572-F6BF2A318492

 MB845388

Fig. 5

###### Diagnosis.

Differing from *S.andina* in the green algal photobiont, the absence of vegetative propagules and the yellow medulla.

**Figure 5.**
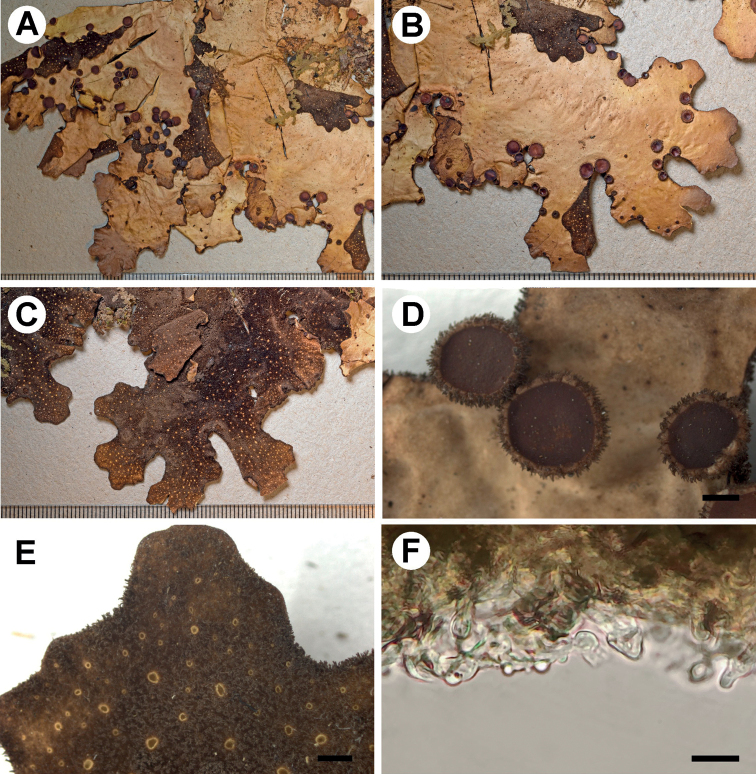
Morphology of *Stictacarrascoensis* (holotype) **A, B** upper surface with marginal and laminal apothecia **C** lower surface **D** apothecia with hirsute margins **E** tomentum, cyphellae and cilia at the lobe margin **F** basal membrane of cyphella with papillae. Scale bars: 1 mm (**A–E**); 10 μm (**F**).

###### Type.

Bolivia. Dept. Cochabamba; Prov. Carrasco, Parque Nacional Carrasco, Meruvia close to Monte Punku, 17°35'06"S, 65°14'54"W, elev. 3283 m, *Podocarpus-Polylepis* forest, Ceja de Monte Inferior (Altimontano), corticolous, 26 Nov 2014, M. Kukwa 15028 (holotype UGDA, isotype LPB).

###### Description.

Primary photobiont a green alga. Stipe absent. Thallus irregular to suborbicular, up to 30 cm diam., moderately branched, with 3–5 branches per 5 cm radius, branching pleurotomus; lobes laciniate to flabellate, interspaced to adjacent, involute, with their apices rounded and plane to involute and their margins entire, not thickened; lobe internodes (3–)6–9(–10) mm long, (3–)9–10(–12) mm broad; thallus coriaceous. Upper surface shallowly scrobiculate to rugose, yellow-brown to light brown, with darker apices in the herbarium, shiny; surface glabrous, without papillae, pruina and weakly maculate; marginal cilia present, brown, about 0.2 mm long, fasciculate. Apothecia abundant, marginal to laminal, dispersed to arranged, sometimes imbricate, subpedicellate, with pronounced invagination on lower side, up to 4 mm diam.; disc orange-brown (young apothecia) to red-brown (older apothecia), very shiny when young; margin crenate, hirsute, with dark brown hairs. Vegetative propagules absent. Lower surface somewhat ridged, yellow to dark brown towards the centre; primary tomentum dense to the margin, thick, thinner towards the margin, spongy, soft, pale to dark brown; secondary tomentum very scarce, up to 25 μm. Rhizines present, about 2 mm, brown with paler tips, fibrillose. Cyphellae 1–10 per cm^2^ towards the thallus centre and 21–40 per cm^2^ towards the margin, scattered, round to irregular, urceolate with wide pore, erumpent to prominent, remaining below the level of the primary tomentum, with the margin raised and involute, brown-coloured, without tomentum or with in the lower part; pore (0.2–)0.3–0.5(–0.6) mm diam.; basal membrane pruinose in the appearance, white to yellow, K– to K+ yellow, C–, KC–, Pd–. Medulla compact, pale yellow to yellow, K+ lemon-yellow, C–, KC–, Pd–. No substances detected by TLC.

Upper cortex paraplectenchymatous, external part orange-brown, 40–60 μm thick, consisting of 6–7 cell layers with cells 5–10 μm diam. (with smaller cells in outside parts of the cortex), their walls 2–3 μm thick and their lumina rounded to isodiametric, 3–10 μm diam., up to 12 μm broad. Photobiont layer 25–35 μm thick, its cells 4–6 μm diam. Medulla 120–150 μm thick, its hyphae 1.5–4.5 μm broad, without or with yellow crystals. Lower cortex paraplectenchymatous, 25–30 μm thick, with 3 cell layers; cells 6–16 μm diam., their walls 1–3 μm thick. Hairs of lower primary tomentum up to 1 mm long, in fascicles up to 12, forming intricate mass in the dense part of tomentum, hyphae unbranched, 5–7 μm broad, septate with free apices. Cyphella cavity up to 250 μm deep; loosely packed cells of basal membrane sometimes with one papilla. Apothecia biatorine, up to 1 mm high, without or very short stipe, about 300 μm long; excipulum up to 100 μm broad, laterally with projecting hairs, simple or in groups, hyphae rarely branched, up to 180 μm long. Hymenium up to 100 μm high; epihymenium up to 25 μm high, pale brown, without gelatinous upper layer. Asci 6–8-spored, ascospores fusiform, 1–3-septate, 26–33 × 7–9 μm.

###### Habitat and distribution.

*Stictacarrascoensis* was collected at a single locality in the Parque Nacional Carrasco in the Department Cochabamba, at an altitude of 3283 m. The specimen grew on the bark of a tree in *Podocarpus-Polylepis* forest.

###### Etymology.

The name refers the type locality.

###### Notes.

*Stictacarrascoensis* is phylogenetically close to *S.andina*, although this relationship is not supported (Fig. 1; Suppl. material 3: Fig. S1). The latter differs by its cyanobacterial photobiont and the formation of isidia and/or phyllidia, not observed in *S.carrascoensis*. The thalli in *S.andina* are smaller (up to 15 cm in diam.), the margins of the lobes are sparsely covered by cilia and the medulla is white to cream, sometimes with yellowish patches and reacts K+ yellow (Moncada et al. 2021a). In *S.carrascoensis*, the medulla is distinctly yellow and reacts K+ lemon-yellow. *Stictaandina* is widespread in South America and, so far, has been confirmed from numerous localities from Brazil, Colombia and Ecuador and also from Costa Rica, Mexico and in Hawaii (Moncada et al. 2014a, b, 2020; Widhelm et al. 2018). Thus far, only one collection of *S.carrascoensis* is known, but it is well-developed and phylogenetically unique, with no close supported relative.

##### ﻿ Sticta catharinae

Taxon classificationFungiPeltigeralesPeltigeraceae

Ossowska, B. Moncada, Kukwa, Flakus, Rodriguez-Flakus & Lücking sp. nov.

E96F2E6F-9F1E-5CD6-99D4-594526142C09

 MB845389

Fig. 6

###### Diagnosis.

Differing from other *Sticta* species in having a stipe, up to 1 cm long, a palmate thallus with abundant, submarginal to laminal apothecia, with the primary tomentum absent in the marginal parts of the thallus and a secondary tomentum present.

**Figure 6.**
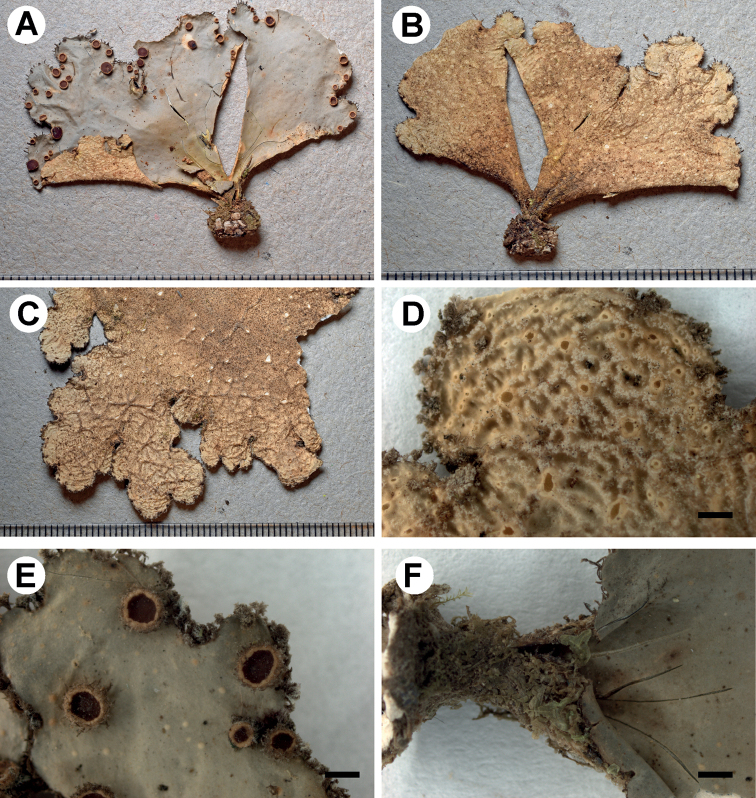
Morphology of *Stictacatharinae* (holotype) **A** upper surface with submarginal to laminal apothecia **B** lower surface **C** lower surface showing venation **D** enlarged part of the lower surface with tomentum and cyphellae **E** apothecia with margin tomentum and hairs and lobe margin with cilia **F** stipe. Scale bars: 1 mm.

###### Type.

Bolivia. Dept. La Paz; Prov. Nur Yungas, Parque Nacional y Área Natural de Manejo Integrado Cotapata, near Urpuma colony, 16°13'20"S, 67°52'34"W, elev. 1989 m, Yungas montane forest, 30 June 2010, A. Flakus 17263 & P. Rodriguez-Flakus (holotype KRAM, isotype LPB).

###### Description.

Primary photobiont cyanobacterial (*Nostoc*). Stipe present, up to 1 cm long. Thallus palmate, up to 10 cm diam., moderately branched, with 3–5 branches per 5 cm radius, branching anisotomous to pleurotomous; lobes suborbicular to flabellate, interspaced to adjacent, involute, with their apices rounded and plane and their margins entire to sinuous, not thickened; lobe internodes (4–)6–15(–20) mm long, (6–)10–10(–20) mm broad; thallus coriaceous. Upper surface smooth to shallowly rugose in some parts, brown-grey, darker in the margins, shiny; surface glabrous, without papillae and pruina, without maculae; marginal cilia abundant, agglutinated to fasciculated, dark brown with pale tips, up to 0.5 mm long. Apothecia abundant, principally submarginal to laminal, dispersed, subpedicellate, sessile, up to 1.5 mm diam.; disc reddish-brown, shiny (in young apothecia) to matt (in older); margin entire to weakly crenate, excipulum hairs few to dense. Vegetative propagules absent. Lower surface folded to distinctly ridged and forming a reticulate pattern especially towards the margins, yellowish-brown to brown towards the centre; primary tomentum scarce, absent in the marginal part of the thallus, fasciculate, soft, golden brown; secondary tomentum present, arachnoid, up to 25 μm. Rhizines absent. Cyphellae 1–20 per cm^2^ towards the thallus centre and 21–40 per cm^2^ towards the margin, scattered, irregular to elongate or rounded, urceolate with narrow to wide pore, prominent, on the same level as the primary tomentum or below, with the margin raised and involute to raised and involute-circinate, brown coloured, with or without tomentum; pore (0.25–)0.5–1(–2) mm diam.; basal membrane smooth, white, K+ yellowish, C–, KC–, Pd–. Medulla loose, white, K± yellowish, C–, KC–, Pd–. No substances detected by TLC.

Upper cortex paraplectenchymatous, 20–50 μm thick, differentiated into two cellular layers, the upper layer consisting of 1–2 layers of cells, with cells 5–6 μm diam., their walls 1–2 μm thick and their lumina rounded to isodiametric, 4–6 μm diam.; the lower layer of cortex 2–3 layers of cells, with cells 8–15 μm diam., their walls 1–2.5 μm thick and their lumina rounded to isodiametric, 3–5 μm diam. Photobiont layer 20–60 μm thick, its cells 6–12 μm diam. Medulla 100–220 μm thick, its hyphae 3–5.5 μm broad, without crystals. Lower cortex paraplectenchymatous, 20–50 μm thick, with 2–4 cell layers; cells 8–17 μm diam., their walls 2–4 μm thick. Hairs of lower primary tomentum up to 220 μm long and 3–5 μm broad, in groups to rarely simple, in fascicles up to 15, hyphae unbranched, septate, with free apices forming a brush-like head. Cyphella cavity up to 120 μm deep; membrane of cells densely packed, cells of basal membrane with 2–4 papillae. Apothecia biatorine, ca. 500 μm high, without distinct stipe; excipulum up to 120 μm broad, without or with projecting hairs, simple to fasciculate. Hymenium 100–120 μm high, hyaline, but K+ yellow; epihymenium ca. 10 μm high, orange-brown, K+ orange intensifying, without gelatinous upper layer. Asci 6–8-spored, ascospores fusiform, 2–4-septate, 31–37 × 8–9 μm.

###### Habitat and distribution.

*Stictacatharinae* is known only from the type locality in Yungas forest in the Department La Paz.

###### Etymology.

The new species is named to honour our late friend and teacher, Polish botanist Dr Katarzyna Żółkoś, for her contributions to the conservation of nature.

###### Notes.

*Stictacatharinae* is the only species amongst those newly described here that is characterised by the presence of a stipe supporting the thallus. Morphologically, this taxon is similar to S.aff.caliginosa D. J. Galloway and the cyanomorph of *S.neopulmonarioides* B. Moncada & Coca, which share the stipe and the palmate thallus. However, in S.aff.caliginosa and *S.neopulmonarioides*, no apothecia are known, whereas vegetative propagules in the form of isidia or phyllidia and lobules are present (Galloway 1997; Moncada 2012; Moncada et al. 2013a). In addition, these taxa differ in the shape of the lobes and the presence of marginal cilia. In S.aff.caliginosa, the lobes are ligulate to flabellate, with their apices rounded to obtuse and with cilia being sparse to absent (Moncada 2012). In contrast, *S.neopulmonarioides* has flabellate lobes with irregular apices, without cilia (Moncada et al. 2013a). *Stictaneopulmonarioides* is widely distributed in Colombia, while S.aff.caliginosa is a rare taxon in that country (Moncada 2012; Moncada et al. 2013a, 2014b).

The new species is closely related to *S.fuliginoides* (Fig. 1; Suppl. material 3: Fig. S1), a morphologically disparate taxon with broad lobes producing laminal isidia (Magain and Sérusiaux 2015). It is also phylogenetically quite distinctive from the latter, with a total of 16 substitutions and nine indels in the ITS (Suppl. material 2: File S1), warranting its formal description, based on a single collection only.

##### ﻿ Sticta pseudoimpressula

Taxon classificationFungiPeltigeralesPeltigeraceae

Ossowska, Kukwa, B. Moncada & Lücking sp. nov.

5C51FB82-FADE-532A-8519-E7C6EE94AF19

 MB845390

Fig. 7

###### Diagnosis.

Differing from *S.impressula* in the presence of imbricately arranged and grouped apothecia with orange-yellow pruina along the margin of the disc, reacting K+ carmine-red and in the presence of a secondary tomentum.

**Figure 7.**
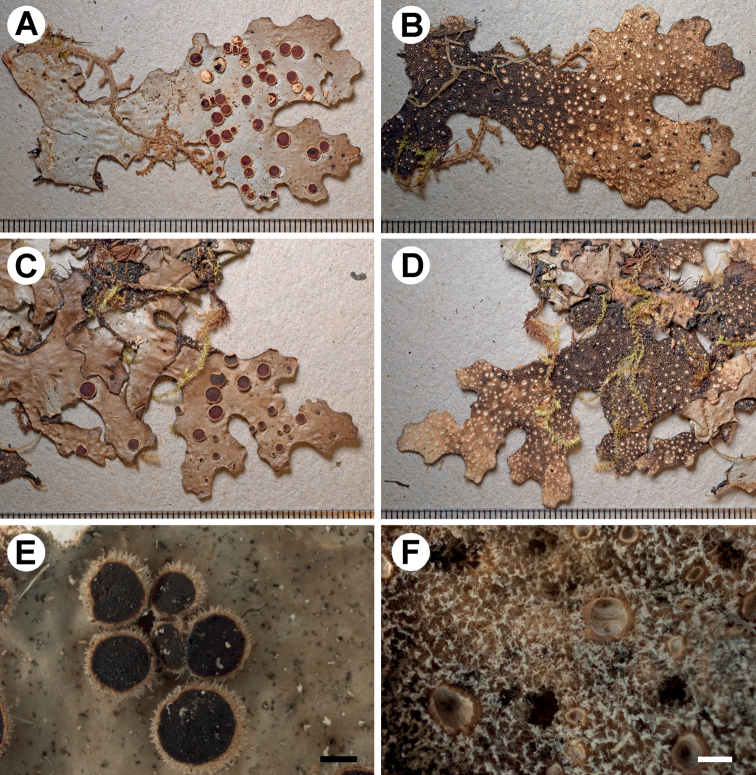
Morphology of *Stictapseudoimpressula* (holotype) **A, C** upper surface with laminal apothecia **B, D** lower surface **E** group of imbricately arranged apothecia with hirsute margins **F** tomentum and cyphellae. Scale bars: 1 mm.

###### Type.

Bolivia. Dept. La Paz; Prov. Franz Tamayo, Área Natural de Manejo Integrado Nacional Apolobamba, near Río Pelechuco, below Pelechuco close to new road to Apolo, 14°46'39"S, 69°00'35"W, elev. 2250 m, lower montane Yungas cloud forest, corticolous, 16 Nov 2014, M. Kukwa 14750 (holotype UGDA, isotype LPB).

###### Description.

Primary photobiont cyanobacterial (*Nostoc*). Stipe absent. Thallus irregular to suborbicular, up to 10 cm diam., moderately branched, with 3–5 branches per 5 cm radius, branching polytomous; lobes laciniate to flabellate, adjacent, plane, with their apices orbicular and revolute to involute and their margins entire to crenate, not thickened; lobe internodes (4–)7–10(–15) mm long, (4–)7–9(–15) mm broad; thallus coriaceous. Upper surface pitted to scrobiculate to rugose towards the centre, yellowish-brown with darker apices in the herbarium, with marginal line in the same colour, shiny; surface glabrous, without papillae and pruina, without maculae; marginal cilia present, abundant, fasciculate, light brown to dark brown, about 0.5 mm. Apothecia abundant, laminal, dispersed, often imbricately arranged and grouped, subpedicellated, with pronounced invagination on lower side, up to 3.5 mm diam.; disc red-brown to brown, sometimes greenish-yellow due to the presence of pruina, shiny; margin entire to crenate and hirsute, with white hairs and orange-yellow pruina. Vegetative propagules absent. Lower surface uneven, beige to dark brown towards the centre; primary tomentum dense to the margin, thick (long), spongy, soft, grey-brown to black with paler tips; secondary tomentum present, but sparse, pubescent, up to 30 μm long. Rhizines sparse, black, up to 0.5 mm. Cyphellae 1–20 per cm^2^ towards the thallus centre and 41–60 per cm^2^ towards the margin, scattered, irregular, cupuliform to urceolate with wide pore, prominent, below the level of the primary tomentum, with the margin erect, cream to brown coloured, without or with tomentum in the lower half; pore (0.5–)0.6–1.3(–2.5) mm diam.; basal membrane pruinose in the appearance, white to pale beige in older part of thallus; K– to K+ pale yellow, C–, KC–, Pd–. Medulla compact, beige-white, K+ yellow, C–, KC–, Pd–. Apothecia margin (ring around disc) and epihymenium K+ carmine-red. No substances detected by TLC, unidentified anthraquinone in apothecia.

Upper cortex paraplectenchymatous, 20–40 μm thick, consisting of 2–3 cell layers with cells 6–22 μm diam. (with smaller cells in outside parts of the cortex), their walls 1–2 μm thick and their lumina rounded to isodiametric, 5–21 μm diam. Photobiont layer 60–110 μm thick, its cells 7–18 μm diam. Medulla 30–100 μm thick, its hyphae 3–5 μm broad, without crystals. Lower cortex paraplectenchymatous, 25–40 μm thick, with 3–4 cell layers; cells 8–18 μm diam., their walls 2–4 μm thick. Hairs of lower primary tomentum up to 1000 μm long, in fascicles of more than 20, hyphae unbranched to rarely branched, septate with flexuous apices. Cyphella cavity 100–125 μm deep; cells of basal membrane loosely packed consisting of cells without papillae or very rarely one. Apothecia biatorine, up to 700 μm high, without distinct stipe; excipulum up to 125 μm broad, laterally with projecting hairs, in groups to rarely simple, up to 0.5 mm, 5–6 μm broad. Hymenium 80–110 μm high; epihymenium 20 μm high, orange, with pigment granules on the top, without gelatinous upper layer. Asci 6–8-spored, ascospores fusiform, 1–3-septate, 28–35 × 8.5–10 μm.

###### Habitat and distribution.

*Stictapseudoimpressula* is an epiphytic species, found in Bolivia at one locality at an altitude of 2250 m in a lower montane Yungas forest in the Department La Paz.

###### Etymology.

The name refers to the similarity in morphology to *Stictaimpressula*.

###### Additional material examined.

Bolivia. Dept. La Paz; Prov. Franz Tamayo, Área Natural de Manejo Integrado Nacional Apolobamba, near Río Pelechuco, below Pelechuco close to new road to Apolo, 14°46'39"S, 69°00'35"W, elev. 2250 m, lower montane Yungas cloud forest, corticolous, 16 Nov 2014, M. Kukwa 14752 (LPB, UGDA).

###### Notes.

*Stictapseudoimpressula* is similar to *S.impressula* (Nyl.) Zahlbr. Both species have a pitted to scrobiculate or rugose upper surface with abundant, laminal apothecia and the lobe margins with abundant, light brown cilia. The tomentum is dense to the margin in the latter (Moncada 2012). However, the two species are not closely related phylogenetically (Fig. 1; Suppl. material 3: Fig. S1): *Stictaimpressula* is clustered in a neighbouring clade with *S.brevior* B. Moncada & Lücking and *S.isidiokunthii*. In contrast, *S.pseudoimpressula* shares a common ancestor with *S.bicellulata*, *S.peltigerella* and *S.sylvatica*. The differences between *S.pseudoimpressula* and *S.bicellulata* are discussed under the latter.

The morphological features that distinguish *S.pseudoimpressula* from *S.impressula* are the moderately-branched thalli and the laciniate to flabellate lobes. The apothecia in *S.pseudoimpressula* are often imbricately arranged and grouped and produced orange-yellow pruina along the disc margins and reacts K+ carmine-red. A secondary tomentum is present in *S.pseudoimpressula*, but absent in *S.impressula*. In addition, the cyphellae in *S.impressula* are rounded to angular and urceolate with a wide pore, erumpent to suprasessile and the margins raised to involute. The latter taxon is widely distributed in Colombia, where it grows at elevations between 1500 and 3800 m (Moncada 2012; Moncada et al. 2014b).

At first sight, *S.pseudoimpressula* can also be confused with *S.brevior*, but that taxon has smaller thalli with abundant apothecia, tomentose margins and the lower surface is undulating, creamy white to light brown (Moncada 2012; Moncada et al. 2013b). *Stictabrevior* is known from Colombia (Moncada et al. 2013b, 2014b).

#### New species from Colombia

##### ﻿ Sticta narinioana

Taxon classificationFungiPeltigeralesPeltigeraceae

B. Moncada, Ossowska & Lücking sp. nov.

28B9319C-0C2F-5544-99C1-A802F8335AAA

 MB845384

Fig. 8

###### Diagnosis.

Differing from *S.aymara* in the predominantly marginal and horizontally projecting isidia, the slightly projecting lower tomentum, giving the impression of marginal cilia, the absence of a secondary tomentum and the more densely arranged cyphellae.

**Figure 8.**
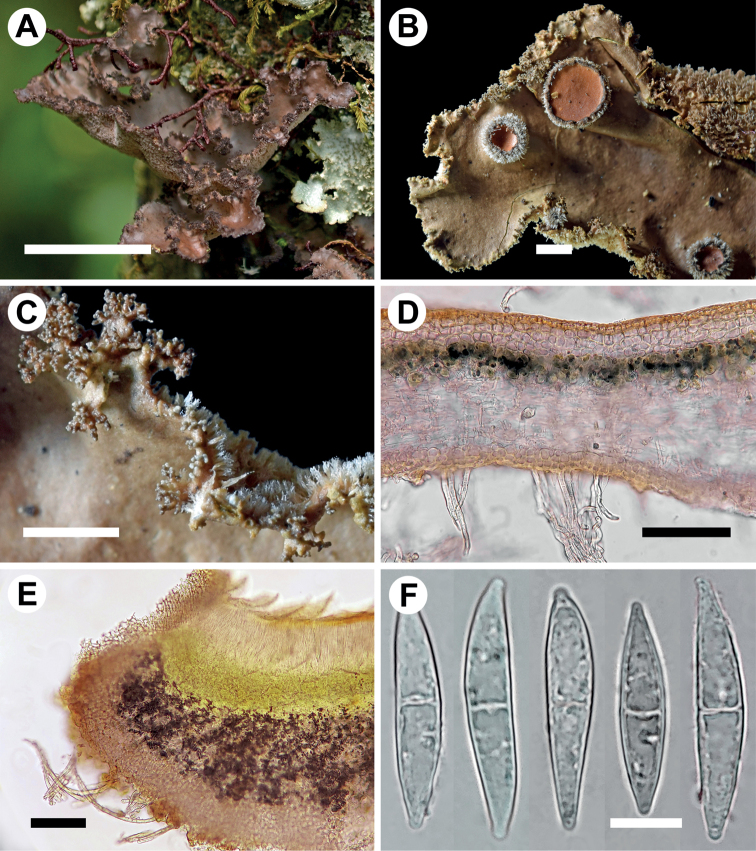
Morphology and anatomy of *Stictanarinioana* (**A** Moncada & Lücking 7525 **B–F** Simijaca et al. 2044) **A** thallus in situ **B** lobe tip with apothecia **C** lobe margin with isidia **D** section through thallus **E** section through apothecium **F** ascospores. Scale bars: 10 mm (**A**); 1 mm (**B, C**); 50 μm (**D**); 100 μm (**E**); 10 μm (**F**).

###### Type.

Colombia. Dept. Nariño; Laguna de la Cocha, Reserva el Encanto Andino, sendero al páramo, 01°04'12.3"N, 77°07'38.1"W, elev. 2810 m, andine forest, epiphytic on tree trunk, 24 Oct 2013, B. Moncada & R. Lücking 7614 (holotype UDBC, isotype B).

###### Description.

Primary photobiont cyanobacterial (*Nostoc*). Stipe absent. Thallus orbicular to irregular, up to 5 cm diam., moderately branched, with 2–5 branches per 5 cm radius, branching pleurotomous; lobes suborbicular to flabellate, interspaced to adjacent, plane to undulate, with their apices rounded to somewhat truncate and revolute and their margins entire to sinuous, not thickened; lobe internodes (3–)5–10 mm long, (3–)5–8(–10) mm broad; thallus subcoriaceous. Upper surface smooth to uneven-rugose towards the centre, greyish-brown when fresh, light to medium yellowish-brown with darker apices in the herbarium, somewhat shiny; surface glabrous, without papillae, pruina or maculae; true marginal cilia absent, but lower tomentum partly projecting beyond the margins and resembling cilia. Apothecia rare to moderately abundant, submarginal, dispersed, subpedicellate, with invagination on lower side, up to 2 mm diam.; disc orange-brown, somewhat shiny; margin densely hirsute, with white hairs. Vegetative propagules present, abundant, in the form of isidia, predominantly marginal, becoming branched and somewhat coralloid, terminally cylindrical, but with the base flattened, more or less obliquely orientated, up to 0.1 mm long and 0.03 mm broad, dark brown and darker than the thallus, shiny. Lower surface somewhat uneven, beige, somewhat darker towards the centre; primary tomentum dense and comparatively thick to the margin, fasciculate, soft, whitish to cream-coloured or pale brownish; secondary tomentum absent, except for the lower sides of the apothecia. Rhizines absent. Cyphellae frequent, 10–20 per cm^2^ towards the thallus centre and 20–50 per cm^2^ towards the margin, dense, rounded to somewhat irregular, urceolate with wide pore, erumpent, remaining below the level of the primary tomentum, with the margin raised and involute, whitish to cream-coloured, with or without tomentum; pore 0.3–1.5 mm diam.; basal membrane ± smooth, white, K–, C–, KC–, Pd–. Medulla compact, cream, K–, C–, KC–, Pd–. No substances detected by TLC.

Upper cortex paraplectenchymatous, 20–40 μm thick, consisting of 2–4 layers of cells 8–15 μm diam. with thin, hyaline walls and one layer of smaller cells with thicker, yellowish-brown walls. Photobiont layer 20–30 μm thick, its cells 5–10 μm diam. Medulla 50–100 μm thick, its hyphae 2.5–5 μm broad, without crystals. Lower cortex paraplectenchymatous, 15–30 μm thick, consisting of 2–3 cell layers; cells 5–10 μm diam., their walls 1–2 μm thick, but lowermost walls much thicker. Hairs of lower primary tomentum 100–200 μm long, in fascicles of 5–20, hyphae simple, septate with partly intertwined apices. Cyphella cavity up to 150 μm deep; cells of basal membrane without papillae or with one papillae. Apothecia biatorine, up to 500 μm high, without distinct stipe; excipulum up to 100 μm broad, laterally with projecting hairs in groups, up to 0.5 mm long and 4–5 μm broad. Hypothecium 60–80 μm high, light yellowish-green. Hymenium 80–110 μm high; epihymenium 15–20 μm high, orange, with pigment granules, without gelatinous upper layer. Asci 8-spored, ascospores fusiform, 1-septate, 35–40 × 7–8 μm.

###### Habitat and distribution.

*Stictanarinioana* is known as epiphyte from two localities of well-preserved (sub-)andine forest in southern Colombia.

###### Etymology.

The epithet honours Antonio Amador José Nariño (y Álvarez del Casal) (1765–1823), one of the critical architects of the independence of Colombia and after whom the Department of Nariño was named.

###### Additional material examined.

Colombia. Dept. Nariño; Laguna de la Cocha, Reserva el Encanto Andino, sendero al páramo, 01°04'12.3"N, 77°07'38.1"W, 2810 m elev., andine forest, epiphytic on tree trunk, 24 October 2013, B. Moncada & R. Lücking 7525 (B, UDBC). Boyacá: Garagoa, Vereda Ciénaga, Valvanera, Reserva Privada El Secreto; 12 June 2014, D. Simijaca et al. 2044 (B, UDBC).

###### Notes.

*Stictanarinioana* is closely related to the Bolivian *S.aymara* described above. Both taxa are cyanobacterial, isidiate species, but *S.aymara* has largely laminal isidia and the lower tomentum is not projecting to resemble cilia. Additionally, the cyphellae are less densely arranged and smaller.

## Supplementary Material

XML Treatment for Sticta amboroensis

XML Treatment for Sticta aymara

XML Treatment for Sticta bicellulata

XML Treatment for Sticta carrascoensis

XML Treatment for Sticta catharinae

XML Treatment for Sticta pseudoimpressula

XML Treatment for Sticta narinioana

## Supplementary materials

Supplementary material 1 Table S1 Data type docx file Explanation note **Tables S1.** Specimens used in this study with the voucher information, references and GenBank accession numbers. Sequences generated during this study are in bold. https://binary.pensoft.net/file/742344This dataset is made available under the Open Database License (http://opendatacommons.org/licenses/odbl/1.0/). The Open Database License (ODbL) is a license agreement intended to allow users to freely share, modify, and use this Dataset while maintaining this same freedom for others, provided that the original source and author(s) are credited.Emilia Anna Ossowska, Bibiana Moncada, Martin Kukwa, Adam Flakus, Pamela Rodriguez-Flakus, Sandra Olszewska, Robert Lücking

Supplementary material 2 File S1 Data type fas file Explanation note Final alignment of the ITS barcoding marker for the studied taxa and the reference sequences. https://binary.pensoft.net/file/742345This dataset is made available under the Open Database License (http://opendatacommons.org/licenses/odbl/1.0/). The Open Database License (ODbL) is a license agreement intended to allow users to freely share, modify, and use this Dataset while maintaining this same freedom for others, provided that the original source and author(s) are credited.Emilia Anna Ossowska, Bibiana Moncada, Martin Kukwa, Adam Flakus, Pamela Rodriguez-Flakus, Sandra Olszewska, Robert Lücking

Supplementary material 3 Figure S1 Data type pdf file Explanation note **Figure S1.** Best-scoring Maximum Likelihood tree of the *Sticta* target clade containing the new species from Bolivia (blue) and Colombia (orange), based on the fungal ITS barcoding marker. Supported clades are thickened and individual support values are indicated. https://binary.pensoft.net/file/742346This dataset is made available under the Open Database License (http://opendatacommons.org/licenses/odbl/1.0/). The Open Database License (ODbL) is a license agreement intended to allow users to freely share, modify, and use this Dataset while maintaining this same freedom for others, provided that the original source and author(s) are credited.Emilia Anna Ossowska, Bibiana Moncada, Martin Kukwa, Adam Flakus, Pamela Rodriguez-Flakus, Sandra Olszewska, Robert Lücking
