# Supplementary material for: ﻿New species of Sticta (lichenised Ascomycota, lobarioid Peltigeraceae) from Bolivia suggest a high level of endemism in the Central Andes
Source: MycoKeys. 2022 Sep 13;92:131–60. doi: 10.3897/mycokeys.92.89960 (PMC9849061; doi:10.3897/mycokeys.92.89960)
Supplement: Supplementary material 1 — Table S1 [file mycokeys-92-131-s001.docx]

**Table S1.** Specimens of *Sticta* used in molecular analysis with locality, voucher information, GenBank accession numbers and list of references. Sequences generated for this study are in bold.

| **Species** | **Voucher and references** | **GenBank number** |
| --- | --- | --- |
| *Sticta andensis* | Colombia, MON0199, Moncada et al. (2014) | KC732547 |
| *Sticta andina* | Brazil, ES3198, Moncada et al. (2020a) | MT936532 |
| *Sticta andina* | Colombia, MON0350, Moncada et al. (2020a) | MT936621 |
| *Sticta andina* | Colombia, MON0812, Widhelm et al. (2018) | MG367411 |
| *Sticta andina* | USA, MON1317, Moncada et al. (2020b) | MT132715 |
| *Sticta andina* | Mexico, MON4149, Moncada et al. (2020a) | MT936763 |
| *Sticta andina* | Costa Rica, BPH15001c, Moncada et al. (2020a) | MT936566 |
| *Sticta andina* | Ecuador, MON4904, Moncada et al. (2020a) | MT936580 |
| *Sticta andina* | Ecuador, MON4912, Moncada et al. (2020a) | MT936581 |
| *Sticta andina* | Colombia, MON2471, Moncada et al. (2020a) | MT936724 |
| *Sticta amboroensis* | Bolivia, Kukwa 9899 | **OP250131** |
| *Sticta amboroensis* | Bolivia, Kukwa 9899a | **OP250132** |
| *Sticta arachnofuliginosa* | Colombia, MON0113, Moncada et al. (2014) | KC732510 |
| *Sticta arachnofuliginosa* | Colombia, MON0143, Moncada et al. (2014) | KC732524 |
| *Sticta arachnofuliginosa* | Colombia, MON0144, Moncada et al. (2014) | KC732525 |
| *Sticta arachnosylvatica* | Colombia, MON0650, Moncada et al. (2014) | KC732718 |
| *Sticta arachnosylvatica* | Colombia, MON0569, Moncada et al. (2014) | KC732772 |
| *Sticta arachnosylvatica* | Colombia, MON0320, Moncada et al. (2014) | KC732588 |
| *Sticta arbuscula* | Colombia, MON100a, Moncada et al. (2014) | KC732502 |
| *Sticta arbuscula* | Colombia, MON100c, Moncada et al. (2014) | KC732504 |
| *Sticta arbuscula* | Colombia, MON0523, Moncada et al. (2014) | KC732741 |
| *Sticta atlantica* | Portugal, LG3858, Magain, Sérusiaux (2015) | KT281737 |
| *Sticta atlantica* | Ireland, LG3747, Magain, Sérusiaux (2015) | KT281734 |
| *Sticta atroandensis* | Colombia, MON0147, Moncada et al. (2014) | KC732528 |
| *Sticta atroandensis* | Colombia, MON0164, Moncada et al. (2014) | KC732532 |
| *Sticta atroandensis* | Colombia, MON0146, Moncada et al. (2014) | KC732527 |
| *Sticta aymara* | Bolivia, Flakus 17220& Rodriguez-Flakus | **OP250125** |
| *Sticta aymara* | Bolivia, Flakus 17220& Rodriguez-Flakus | **OP250126** |
| *Sticta beauvoisii* | Colombia, MON0625, Moncada et al. (2014) | KC732707 |
| *Sticta beauvoisii* | USA, McDonald et al. (2003) | AY173378 |
| *Sticta beauvoisii* | USA, McDonald et al. (2003) | AY173372 |
| *Sticta bicellulata* | Bolivia, Kukwa 14859 | **OP250129** |
| *Sticta bicellulata* | Bolivia, Kukwa 14863 | **OP250130** |
| *Sticta borinquensis* | Puerto Rico, DNA14658, Mercado-Díaz et al. (2020) | MN065856 |
| *Sticta borinquensis* | Puerto Rico, MON0574, Widhelm et al. (2018) | MG367397 |
| *Sticta borinquensis* | Puerto Rico, DNA14660, Mercado-Díaz et al. (2020) | MN065857 |
| *Sticta brevior* | Colombia, MON0175, Moncada et al. (2014) | KC732535 |
| *Sticta brevior* | Colombia, MON0328, Moncada et al. (2014) | KC732596 |
| *Sticta brevior* | Colombia, MON0093, Moncada et al. (2014) | KC732499 |
| *Sticta canariensis* | Spain, LG1333, Magain, Sérusiaux (2015) | KT281700 |
| *Sticta canariensis* | Ireland, LG3741, Magain, Sérusiaux (2015) | KT281733 |
| *Sticta canariensis* | Spain, Cornejo et al. (2009) | DQ419944 |
| *Sticta carolinensis* | USA, McDonald et al. (2003) | AY173380 |
| *Sticta carolinensis* | USA, McDonald et al. (2003) | AY173381 |
| *Sticta catharinae* | Bolivia, Flakus 17263 & Rodriguez-Flakus | **OP250134** |
| *Sticta catharinae* | Bolivia, Flakus 17263a & Rodriguez-Flakus | **OP250135** |
| *Sticta carrascoensis* | Bolivia, Kukwa 15028 | **OP250133** |
| *Sticta carolinensis* | USA, McDonald et al. (2003) | AY173379 |
| *Sticta ciliata* | Costa Rica, MON0724, Moncada et al. (2020b) | MT132622 |
| *Sticta ciliata* | Spain, LG3830, Magain, Sérusiaux (2015) | KT281719 |
| *Sticta ciliata* | Colombia, MON1011, Moncada et al. (2020b) | MT132633 |
| *Sticta ciliata* | Colombia, MON0817, Moncada et al. (2020b) | MT132628 |
| *Sticta ciliata* | Spain, LG2751, Magain, Sérusiaux (2015) | KT281712 |
| *Sticta ciliata* | Spain, LG3406, Magain, Sérusiaux (2015) | KT281713 |
| *Sticta ciliata* | France, LG3542, Magain, Sérusiaux (2015) | KT281714 |
| *Sticta ciliata* | Portugal, LG3099, Magain, Sérusiaux (2015) | KT281715 |
| *Sticta ciliata* | Ireland, LG3781, Magain, Sérusiaux (2015) | KT281716 |
| *Sticta ciliata* | USA, MON1217, Moncada et al. (2020b) | MT132672 |
| *Sticta cometia* | Colombia, MON0413, Moncada et al. (2014) | KC732642 |
| *Sticta cometia* | Colombia, MON0396, Moncada et al. (2014) | KC732627 |
| *Sticta cometia* | Colombia, MON0412, Moncada et al. (2014) | KC732641 |
| *Sticta cometiella* | Colombia, MON0339, Moncada et al. (2014) | KC732606 |
| *Sticta cometiella* | Colombia, MON0127, Moncada et al. (2014) | KC732516 |
| *Sticta cometiella* | Colombia, MON0237, Moncada et al. (2014) | KC732556 |
| *Sticta dilatata* | Colombia, MON0420, Moncada et al. (2014) | KC732648 |
| *Sticta dilatata* | Colombia, MON0419, Moncada et al. (2014) | KC732647 |
| *Sticta duplolimbata* | Rwanda, LG0919, Magain, Sérusiaux (2015) | KT281696 |
| *Sticta duplolimbata* | Taiwan, | AB245117 |
| *Sticta fragilinata* | USA, McDonald et al. (2003) | AY173383 |
| *Sticta fuliginoides* | Colombia, MON0640, Moncada et al. (2014) | KC732709 |
| *Sticta fuliginoides* | USA, McDonald et al. (2003) | AY173388 |
| *Sticta fuliginoides* | France, LG1421, Magain, Sérusiaux (2015) | KT281701 |
| *Sticta fuliginoides* | UK, DNA4907b, Moncada et al. (2014) | KC732454 |
| *Sticta fuliginoides* | Spain, LG3012, Magain, Sérusiaux (2015) | KT281722 |
| *Sticta fuliginosa* | Spain, LG3010, Magain, Sérusiaux (2015) | KT281721 |
| *Sticta fuliginosa* | Portugal, LG3100, Magain, Sérusiaux (2015) | KT281704 |
| *Sticta fuliginosa* | Ireland, LG3729, Magain, Sérusiaux (2015) | KT281731 |
| *Sticta fuliginosa* | France, LG3537, Magain, Sérusiaux (2015) | KT281727 |
| *Sticta fuliginosa* | UK, LG00S9, Magain, Sérusiaux (2015) | KT281739 |
| *Sticta fuliginosa* | France, LG0989, Magain, Sérusiaux (2015) | KT281698 |
| *Sticta fuliginosa* | Japan, Takahashi et al. (2006) | AB239345 |
| *Sticta fuliginosa* | Rwanda, LG1611, Magain, Sérusiaux (2015) | KT281702 |
| *Sticta fuliginosa* | South Africa, LG1952, Magain, Sérusiaux (2015) | KT281703 |
| *Sticta fuliginosa* | USA, MON1293, Widhelm et al. (2018) | MG367432 |
| *Sticta fuscotomentosa* | Colombia, Moncada et al. (2014) | KC732661 |
| *Sticta gallowayana* | Colombia, MON0649, Moncada et al. (2014) | KC732717 |
| *Sticta gallowayana* | Colombia, MON0103, Moncada et al. (2014) | KC732496 |
| *Sticta gallowayana* | Colombia, MON0108, Moncada et al. (2014) | KC732507 |
| *Sticta globulifuliginosa* | Colombia, MON5464, Moncada et al. (2014) | KC732601 |
| *Sticta globulifuliginosa* | Colombia, MON0306, Moncada et al. (2014) | KC732576 |
| *Sticta globulifuliginosa* | Colombia, MON0318, Moncada et al. (2014) | KC732586 |
| *Sticta gyalocarpa* | Colombia, MON0326, Moncada et al. (2014) | KC732594 |
| *Sticta gyalocarpa* | Colombia, MON0307, Moncada et al. (2014) | KC732577 |
| *Sticta gyalocarpa* | Colombia, MON0004, Moncada et al. (2014) | KC732455 |
| *Sticta hirsutofuliginosa* | Colombia, MON0348, Moncada et al. (2014) | KC732612 |
| *Sticta hirsutofuliginosa* | Colombia, MON0346, Moncada et al. (2014) | KC732610 |
| *Sticta hirsutofuliginosa* | Colombia, MON0099, Moncada et al. (2014) | KC732501 |
| *Sticta hirta* | Colombia, MON0117, Moncada et al. (2014) | KC732513 |
| *Sticta hirta* | Colombia, MON0151, Moncada et al. (2014) | KC732529 |
| *Sticta hirta* | Colombia, MON0153, Moncada et al. (2014) | KC732563 |
| *Sticta humboldtii* | Colombia, MON0614, Moncada et al. (2014) | KC732703 |
| *Sticta humboldtii* | Colombia, MON0615, Moncada et al. (2014) | KC732704 |
| *Sticta humboldtii* | Colombia, MON0613, Moncada et al. (2014) | KC732702 |
| *Sticta impressula* | Colombia, MON0414, Moncada et al. (2014) | KC732643 |
| *Sticta impressula* | Colombia, MON0387, Moncada et al. (2014) | KC732620 |
| *Sticta impressula* | Colombia, MON0416, Moncada et al. (2014) | KC732644 |
| *Sticta isidiokunthii* | Colombia, MON0024, Moncada et al. (2014) | KC732462 |
| *Sticta isidiokunthii* | Colombia, MON0471, Moncada et al. (2014) | KC732685 |
| *Sticta isidiokunthii* | Colombia, MON0469, Moncada et al. (2014) | KC732683 |
| *Sticta laciniata* | Costa Rica, MON0684, Widhelm et al. (2018) | MG367399 |
| *Sticta laevis* | Colombia, Widhelm et al. (2018) | MG367409 |
| *Sticta latior* | Brazil, MON0273, Moncada et al. (2014) | KC732568 |
| *Sticta leucoblepharis* | Colombia, MON0445, Moncada et al. (2014) | KC732664 |
| *Sticta leucoblepharis* | Colombia, MON0331, Moncada et al. (2014) | KC732599 |
| *Sticta leucoblepharis* | Colombia, MON0329, Moncada et al. (2014) | KC732597 |
| *Sticta limbata* | USA, DNA10049, Widhelm et al. (2018) | MG367378 |
| *Sticta limbata* | Portugal, LG3868, Magain, Sérusiaux (2015) | KT281711 |
| *Sticta limbata* | USA, MON1262, Widhelm et al. (2018) | MG367428 |
| *Sticta limbata* | France, LG3544, Magain, Sérusiaux (2015) | KT281728 |
| *Sticta limbata* | Spain, LG2749, Magain, Sérusiaux (2015) | KT281708 |
| *Sticta limbata* | UK, LG2690, Magain, Sérusiaux (2015) | KT281707 |
| *Sticta lobarioides* | Colombia, MON0236, Moncada et al. (2014) | KC732555 |
| *Sticta lobarioides* | Colombia, MON0404, Moncada et al. (2014) | KC732634 |
| *Sticta lobulata* | Colombia, MON0035, Moncada et al. (2014) | KC732471 |
| *Sticta lobulata* | Colombia, MON0496, Moncada et al. (2014) | KC732727 |
| *Sticta lobulata* | Colombia, MON0060, Moncada et al. (2014) | KC732482 |
| *Sticta lumbschiana* | Colombia, MON0308, Moncada et al. (2014) | KC732578 |
| *Sticta lumbschiana* | Colombia, MON0524, Moncada et al. (2014) | KC732742 |
| *Sticta lumbschiana* | Colombia, MON0567, Moncada et al. (2014) | KC732771 |
| *Sticta macrofuliginosa* | Colombia, MON0533, Moncada et al. (2014) | KC732747 |
| *Sticta macrothallina* | Colombia, MON0431b, Moncada et al. (2014) | KC732656 |
| *Sticta macrothallina* | Colombia, MON0408, Moncada et al. (2014) | KC732637 |
| *Sticta macrothallina* | Colombia, MON0431a, Moncada et al. (2014) | KC732655 |
| *Sticta maculofuliginosa* | Colombia, MON121b, Moncada et al. (2014) | KC732515 |
| *Sticta maculofuliginosa* | Colombia, MON0122, Moncada et al. (2014) | KC732561 |
| *Sticta maculofuliginosa* | Colombia, MON121a, Moncada et al. (2014) | KC732514 |
| *Sticta minutula* | Colombia, MON0315, Moncada et al. (2014) | KC732583 |
| *Sticta minutula* | Colombia, MON0114, Moncada et al. (2014) | KC732511 |
| *Sticta minutula* | Colombia, MON0482, Moncada et al. (2014) | KC732719 |
| *Sticta narinioana* | Colombia, MON2696, Simijaca et al. 2044 | **OP244962** |
| *Sticta narinioana* | Colombia, MON2280, Moncada & Lücking 7614 | **OP244963** |
| *Sticta narinioana* | Colombia, MON2272, Moncada & Lücking 7525 | **OP244961** |
| *Sticta neopulmonarioides* | Colombia, MON0406, Moncada et al. (2014) | KC732636 |
| *Sticta neopulmonarioides* | Colombia, MON0403, Moncada et al. (2014) | KC732633 |
| *Sticta neopulmonarioides* | Colombia, MON0397, Moncada et al. (2014) | KC732628 |
| *Sticta papillata* | Colombia, MON0832, Widhelm et al. (2018) | MG367414 |
| *Sticta papillata* | Colombia, MON0215, Moncada et al. (2014) | KC732551 |
| *Sticta papillata* | Colombia, MON0216, Moncada et al. (2014) | KC732552 |
| *Sticta parahumboldtii* | Colombia, MON0209, Moncada et al. (2014) | KC732550 |
| *Sticta parahumboldtii* | Colombia, MON0294, Moncada et al. (2014) | KC732573 |
| *Sticta peltigerella* | Colombia, MON0814, Moncada et al. (2020) | MT132627 |
| *Sticta peltigerella* | Colombia, MON0887, Moncada et al. (2020) | MT132629 |
| *Sticta peltigerella* | Colombia, MON4410, Moncada et al. (2020) | MT132763 |
| *Sticta phyllidiofuliginosa* | Colombia, MON0559, Moncada et al. (2014) | KC732764 |
| *Sticta phyllidiofuliginosa* | Colombia, MON0109, Moncada et al. (2014) | KC732508 |
| *Sticta phyllidiofuliginosa* | Colombia, MON0088, Moncada et al. (2014) | KC732495 |
| *Sticta phyllidiokunthii* | Colombia, MON0470, Moncada et al. (2014) | KC732684 |
| *Sticta phyllidiokunthii* | Colombia, MON0325, Moncada et al. (2014) | KC732593 |
| *Sticta phyllidiokunthii* | Colombia, MON0534, Moncada et al. (2014) | KC732748 |
| *Sticta plumbeociliata* | Colombia, MON0560, Moncada et al. (2014) | KC732765 |
| *Sticta plumbeociliata* | Colombia, MON0563, Moncada et al. (2014) | KC732767 |
| *Sticta pseudohumboldtii* | Colombia, MON0190, Moncada et al. (2014) | KC732543 |
| *Sticta pseudohumboldtii* | Colombia, MON0517, Moncada et al. (2014) | KC732737 |
| *Sticta pseudohumboldtii* | Colombia, MON0515, Moncada et al. (2014) | KC732735 |
| *Sticta pseudoimpressula* | Bolivia, Kukwa 14750 | **OP250127** |
| *Sticta pseudoimpressula* | Bolivia, Kukwa 14752 | **OP250128** |
| *Sticta pseudolobaria* | Colombia, MON0428, Moncada et al. (2014) | KC732653 |
| *Sticta pseudolobaria* | Colombia, MON0421, Moncada et al. (2014) | KC732649 |
| *Sticta pseudolobaria* | Colombia, MON0425, Moncada et al. (2014) | KC732650 |
| *Sticta pulmonarioides* | Colombia, MON0402, Moncada et al. (2014) | KC732632 |
| *Sticta pulmonarioides* | Colombia, MON0435, Moncada et al. (2014) | KC732660 |
| *Sticta pulmonarioides* | Colombia, MON0434, Moncada et al. (2014) | KC732659 |
| *Sticta rhizinata* | Colombia, MON0079, Moncada et al. (2014) | KC732492 |
| *Sticta rhizinata* | Colombia, MON0186, Moncada et al. (2014) | KC732541 |
| *Sticta rhizinata* | Colombia, MON0351, Moncada et al. (2014) | KC732613 |
| *Sticta scabrosa* | Brazil, MON5132, Moncada et al. (2020a) | MT936598 |
| *Sticta scabrosa* | Brazil, MON5134, Moncada et al. (2020a) | MT936600 |
| *Sticta scabrosa* | Colombia, MON2323, Moncada et al. (2020a) | MT936720 |
| *Sticta scabrosa* | Puerto Rico, MON2503, Moncada et al. (2020a) | MT936726 |
| *Sticta scabrosa* | Colombia, MON0906, Moncada et al. (2020a) | MT936648 |
| *Sticta scabrosa* | Colombia, MON2560, Moncada et al. (2020a) | MT936729 |
| *Sticta scabrosa* | Galapagos, MON2772, Moncada et al. (2020a) | MT936736 |
| *Sticta scabrosa* | Mexico, MON2417, Moncada et al. (2020a) | MT936722 |
| *Sticta scabrosa* | Colombia, MON0142, Moncada et al. (2020a) | MT936610 |
| *Sticta scabrosa* | Argentina, MON1126, Moncada et al. (2020a) | MT936666 |
| *Sticta schizophylliza* | Puerto Rico, DNA14861, Mercado-Díaz et al. (2020) | MN065832 |
| *Sticta schizophylliza* | Puerto Rico, DNA14857, Mercado-Díaz et al. (2020) | MN065830 |
| *Sticta schizophylliza* | Puerto Rico, MON0572, Moncada et al. (2014) | KC732773 |
| *Sticta sublimbata* | Japan, Takahashi et al. (2006) | AB245118 |
| *Sticta sublimbata* | Japan, Takahashi et al. (2006) | AB245123 |
| *Sticta sublimbata* | France, LG1038, Magain, Sérusiaux (2015) | KT281699 |
| *Sticta subscrobiculata* | Colombia, MON0204, Moncada et al. (2014) | KC732549 |
| *Sticta subscrobiculata* | Colombia, MON0499, Moncada et al. (2014) | KC732728 |
| *Sticta sylvatica* | UK, LG3723, Magain, Sérusiaux (2015) | KT281730 |
| *Sticta sylvatica* | France, LG3536, Magain, Sérusiaux (2015) | KT281726 |
| *Sticta sylvatica* | Colombia, MON0313, Moncada et al. (2014) | KC732581 |
| *Sticta tomentosa* | Colombia, MON0442, Moncada et al. (2014) | KC732663 |
| *Sticta tomentosa* | Colombia, MON2175, Moncada et al. (2020b) | MT132745 |
| *Sticta tomentosa* | Ecuador, MON4891, Moncada et al. (2020b) | MT132764 |
| *Sticta viviana* | Colombia, MON0345, Moncada et al. (2014) | KC732609 |
| *Sticta viviana* | Colombia, MON0462, Moncada et al. (2014) | KC732680 |
| *Sticta viviana* | Colombia, MON0472, Moncada et al. (2014) | KC732686 |
| *Sticta weigelli* | Colombia, MON0062, Moncada et al. (2014) | KC732484 |
| *Sticta weigelli* | Bolivia, Ossowska (2021) | MZ292977 |
| *Sticta weigelli* | Bolivia, Ossowska (2021) | MZ292976 |
| *Sticta weigelli* | Colombia, MON0061, Moncada et al. (2014) | KC732483 |
| *Sticta weigelli* | Puerto Rico, DNA14945, Mercado-Díaz et al. (2020) | MN065896 |
| *Sticta weigelli* | Puerto Rico, DNA14532, Mercado-Díaz et al. (2020) | MN065897 |

**References**

Cornejo C, Chabanenko S, Scheidegger C (2009) Phylogenetic analysis indicates transitions from vegetative to sexual reproduction in the *Lobaria* *retigera* group (Lecanoromycetidae, Ascomycota). Lichenologist 41 (3): 275–284. https://doi.org/10.1017/S0024282909006240

Magain N, Sérusiaux E (2015) Dismantling the treasured flagship lichen *Sticta fuliginosa* (Peltigerales) into four species in Western Europe. Mycological Progress 14: 97. https://doi.org/10.1007/s11557-015-1109-0

McDonald T, Miadlikowska J, Lutzoni F (2003) The lichen genus *Sticta* in the Great Smoky Mountains: A phylogenetic study of morphological, chemical, and molecular data. The Bryologist 106(1): 61–79. https://doi.org/10.1639/0007-2745(2003)106[0061:TLGSIT]2.0.CO;2

Mercado-Díaz JA, Lücking R, Moncada B, Widhelm TJ, Lumbsch HT (2020) Elucidating species richness in lichen fungi: The genus *Sticta* (Ascomycota: Peltigeraceae) in Puerto Rico. Taxon 69 (5): 1–41. https://doi.org/10.1002/tax.12320

Moncada B, Lücking R, Suárez A (2014) Molecular phylogeny of the genus *Sticta* (lichenized Ascomycota: Lobariaceae) in Colombia. Fungal Diversity 64: 205–231. https://doi.org/10.1007/s1322 5-013-0230-0

Moncada B, Mercado-Díaz JA, Magain N, Hodkinson BP, Smith CW, Bungartz F, Pérez-Pérez R-E, Gumboski E, Sérusiaux E, Lumbsch HT, Lücking R (2020a) Phylogenetic diversity of two geographically overlapping lichens: isolation by distance, environment, or fragmentation? Journal of Biogeography 48(3): 676–689. https://doi.org/10.1111/jbi.14033

Moncada B, Lücking R, Lumbsch HT (2020b) Rewriting the evolutionary history of the lichen genus *Sticta* (Ascomycota: Peltigeraceae subfam. Lobarioideae) in the Hawaiian islands. Plant and Fungal Systematics 65(1): 95–119. https://doi.org/10.35535/pfsyst-2020-0005

Ossowska EA (2021) First records of *Sticta weigelii* s.str. from Bolivia confirmed by molecular data. Folia Cryptogamica Estonica 58: 65–72. https://doi.org/10.12697/fce.2021.58.09

Takahashi K, Wang LS, Tsubota H, Deguchi H (2006) Photosymbiodemes *Sticta wrightii* and *Dendriscocaulon* sp. (Lichenized Ascomycota) from Yunnan, China. Journal- Hattori Botanical Laboratory 100: 783–796.

Widhelm TJ, Bertoletti FR, Asztalos MJ, Mercado-Díaz JA, Huang J-P., Moncada B, Lücking R, Magain N, Sérusiaux E, Goffinet B, Crouch N, Mason-Gamer R, Lumbsch HT (2018) Oligocene origin and drivers of diversification in the genus *Sticta* (Lobariaceae, Ascomycota). Molecular Phylogenetics and Evolution 126: 58–73. https://doi.org/10.1016/j.ympev.2018.04.006
