# Supplementary figures and images for: ﻿New species of Sticta (lichenised Ascomycota, lobarioid Peltigeraceae) from Bolivia suggest a high level of endemism in the Central Andes
Source: MycoKeys. 2022 Sep 13;92:131–60. doi: 10.3897/mycokeys.92.89960 (PMC9849061; doi:10.3897/mycokeys.92.89960)

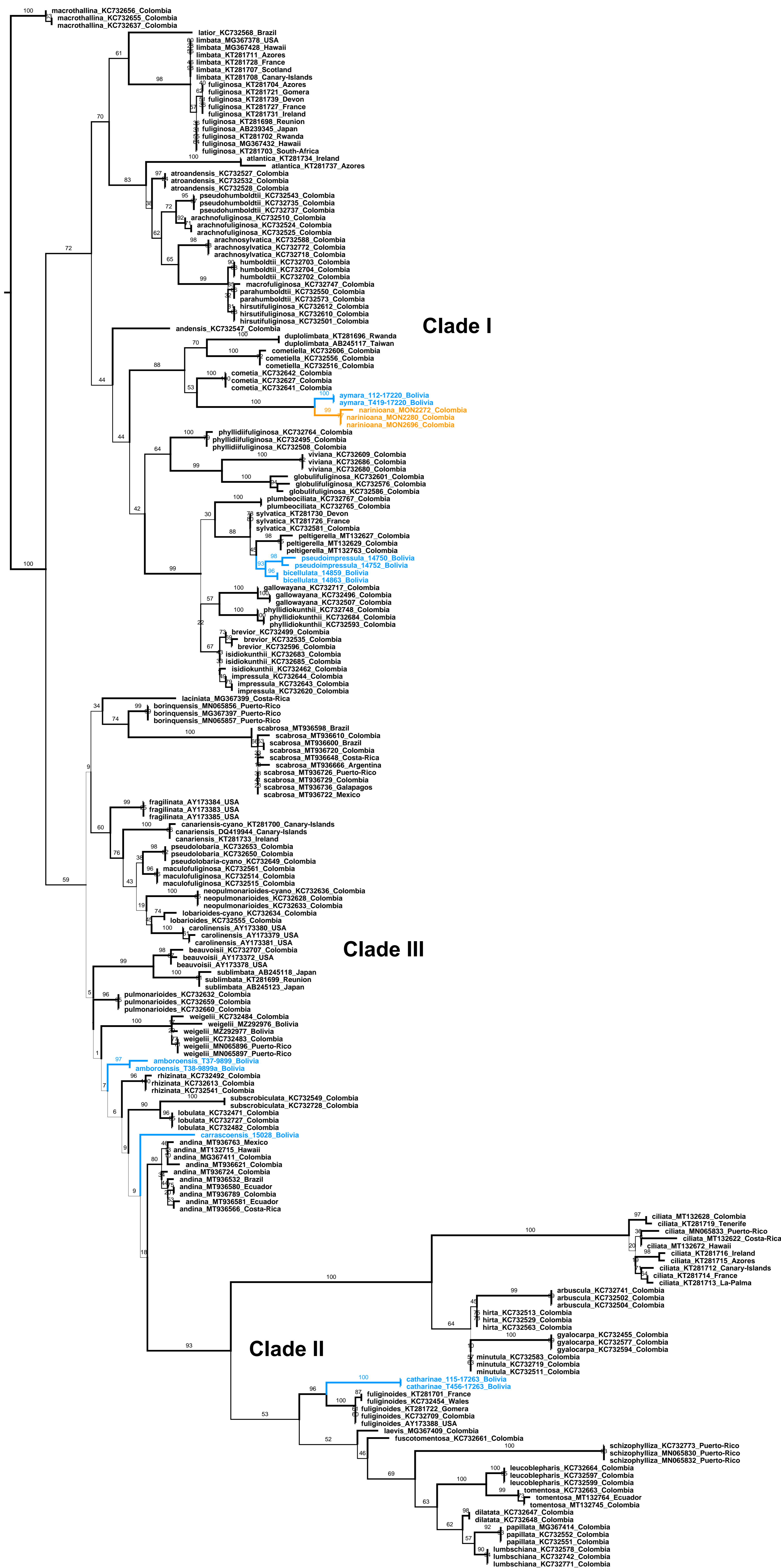

Supplement: Supplementary material 3 — Figure S1 [file mycokeys-92-131-s003.pdf]
